# Multi-omics and network pharmacology approaches reveal the mechanism of action of KeKe tablet against post-infectious cough

**DOI:** 10.1186/s13020-025-01199-7

**Published:** 2025-10-04

**Authors:** Mengyue Su, Jiahe Zhang, Shukun Wang, Weixuan Chen, Minling Lai, Lihua Peng, Yanling Liang, Yu Feng, Hua Zhou, Weilin Qiao, Peng Sun

**Affiliations:** 1 School of Pharmacy, Shandong University of Traditional Chinese Medicine, Jinan, People’s Republic of China; 2 Innovative Chinese Medicine Research Institute, Zhongshan Zhongzhi Pharmaceutical Co., Ltd, Zhongshan, People’s Republic of China; 3 Guangdong Provincial Hospital of Chinese Medicine, Guangdong Provincial Academy of Chinese Medical Sciences, State Key Laboratory of Dampness, Syndrome of Chinese Medicine, Second Affiliated Hospital of Guangzhou University of Chinese Medicine, Guangzhou University of Chinese Medicine, Guangzhou, People’s Republic of China; 4 Innovative Institute of Chinese Medicine and Pharmacy, Shandong University of Traditional Chinese Medicine, Jinan, People’s Republic of China

**Keywords:** Keke tablets, Post infectious cough, Transcriptomics, Proteomics, Inflammation

## Abstract

**Background:**

Post-infectious cough (PIC) refers to a persistent cough lasting longer than three consecutive weeks following an infection, despite the resolution of other symptoms and normal examination results. This common respiratory disorder significantly impacts patients' quality of life due to the ongoing coughing. The Keke tablet (KKP), an herbal medicine preparation, is frequently used as an over-the-counter (OTC) treatment for respiratory conditions. However, the mechanisms underlying KKP's efficacy are not yet fully understood.

**Objective:**

This study aims to evaluate the therapeutic effects of KKP in treating PIC and to explore its potential mechanisms of action.

**Methods:**

Rat models of post-infectious cough (PIC) were established through exposure to cigarette smoke, capsaicin nebulization, and intranasal instillation of lipopolysaccharide, followed by intragastric administration of Keke tablets (KKP). Hematoxylin and eosin (HE) staining assessed the morphology of trachea and lung tissues. Cytokine levels in serum and lung tissues were measured using ELISA kits. Immunofluorescence techniques were utilized to visualize neurotransmitters in airway and lung tissues. Network pharmacology, transcriptomic and proteomic analyses were conducted to explore the underlying mechanisms of KKP, while Western blotting estimated the expression levels of proteins involved in relevant pathways.

**Results:**

KKP significantly improved the pathological damage in airways and lung tissues and enhanced lung function in rats with PIC. Treatment with KKP resulted in decreased concentrations of inflammatory mediators in both serum and lung tissues, alleviating inflammatory responses. Additionally, KKP effectively reversed neurogenic inflammation associated with PIC by enhancing neutral endopeptidase activity and reducing the levels of substance P and calcitonin gene-related peptide. Network pharmacology, transcriptomics and proteomics analyses indicated that KKP exerted its therapeutic effects by regulating inflammation-related pathways such as MAPK/NF-κB signaling pathway. Western blot analysis further confirmed that KKP activated this pathway, evidenced by reduced expression of p65-NF-κB and p-38 MAPK following KKP administration.

**Conclusion:**

Our findings highlighted the clinical potential of Keke Tablet in the treatment of post-infectious cough, and confirmed that Keke tablet improved cough symptoms by alleviating inflammatory response. MAPK/NF-κB signaling pathway is one of the potential pathways of Keke tablet in the treatment of post-infectious cough.

**Supplementary Information:**

The online version contains supplementary material available at 10.1186/s13020-025-01199-7.

## Induction

A cough persisting for more than three consecutive weeks following an acute upper respiratory tract infection is termed Post-Infectious Cough (PIC), diagnosed when the infection symptoms have resolved and chest X-rays are normal [7]. This condition is common in respiratory clinics, with causes including air pollution, cold exposure, viral or bacterial infections after excessive fatigue, and various physical and chemical stimuli [8, 24]. The pathogenesis of cough induced by respiratory tract infection involves complex interactions among multiple factors and pathways. Primarily, respiratory viruses can directly damage airway epithelial cells, disrupt tight junctions and compromise the epithelial barrier function, thereby increasing airway sensitivity to external stimuli [20]. Concurrently, the innate immune system is activated via pattern recognition receptors, initiating downstream signaling cascades that promote the release of pro-inflammatory mediators and recruit inflammatory cells, which in turn exacerbate airway inflammation and expose sensory nerve endings [52]. At the neural level, viral and other noxious stimuli activate transient receptor potential (TRP) channels located on vagal C fibers, triggering action potential transmission that induces the cough reflex [48, 66]. Additionally, due to epithelial injury and impaired degradation, neuropeptides released by sensory nerve endings are sustained, further stimulating cough-related receptors indirectly by acting on effector cell receptors. Persistent inflammation may also lead to airway remodeling, such as basement membrane thickening and smooth muscle hyperplasia, contributing to airway hyperresponsiveness and increased airway resistance [56]. At the central level, peripheral inflammatory mediators may induce central sensitization, lowering the threshold for sensory input and amplifying cough responses [15]. Collectively, these mechanisms contribute to the onset and persistence of post-infectious cough. While PIC is generally self-limiting and not typically associated with severe frailty or mortality, the persistent cough can significantly impair patients'quality of life, often prompting medical consultation.

For modern medicine, Traditional Chinese Medicine (TCM) offers a wealth of potential resources. Several TCM formulations for PIC treatment have been developed and are in clinical use. For example, Suhuang antitussive capsules have shown efficacy in alleviating PIC in mice through the AhR-Nrf2 pathway [45]. In guinea pigs, Xingbei antitussive granules regulate tryptase/PAR2/TRPV1 pathways to ease cough hypersensitivity [43]. Qinbai Qingfei Concentrated Pellets have been found to reverse neurogenic inflammation induced by PIC by modulating Neutral endopeptidase (NEP) activity and reducing the expression of substance P (SP) and calcitonin gene-related peptide (CGRP) [30]. Zhisou powder demonstrates anti-inflammatory effects on the airways and alleviates cough hypersensitivity in PIC model mice [75].

Keke Tablet, a TCM formulation widely used for respiratory diseases, is available over the counter and offers antitussive, antiasthmatic, and expectorant properties. Its main ingredients include Ephedrae herba (Mahuang), Papaveris Pericarpium (Yingsuke), Glycyrrhizae Radix Et Rhizoma (Gancao), Armeniacae Semen Amarum (Kuxingren), Raphani Semen (Laifuzi), Platycodonis Radix (Jiegeng), and Gypsum Fibrosum (Shigao). Derived from the Maxing-Shigan Decoction—an established prescription by Zhang Zhongjing for treating lung heat and asthma—Keke Tablet has been reported effective against pneumonia, pulmonary influenza virus infection, chronic obstructive pulmonary disease, and COVID-19 [9, 27, 42]. Modifications to the original decoction enhance its efficacy in relieving cough and asthma symptoms while broadening its indications. Studies indicate that Keke Tablet exhibits significant anti-inflammatory, antitussive, and antiasthmatic effects with a strong safety profile, particularly in treating wind-cold and phlegm-dampness syndromes associated with chronic bronchitis [40, 82]. Furthermore, it shows promise in improving conditions in aged rats with chronic obstructive pulmonary disease [34]. However, there is currently no research on the evaluation of the efficacy and exploration of the mechanism of action of Keke Tablet in the treatment of post-infectious cough. So, it is necessary to study the pharmacodynamics and mechanism of Keke tablets.

**Fig. 1:**
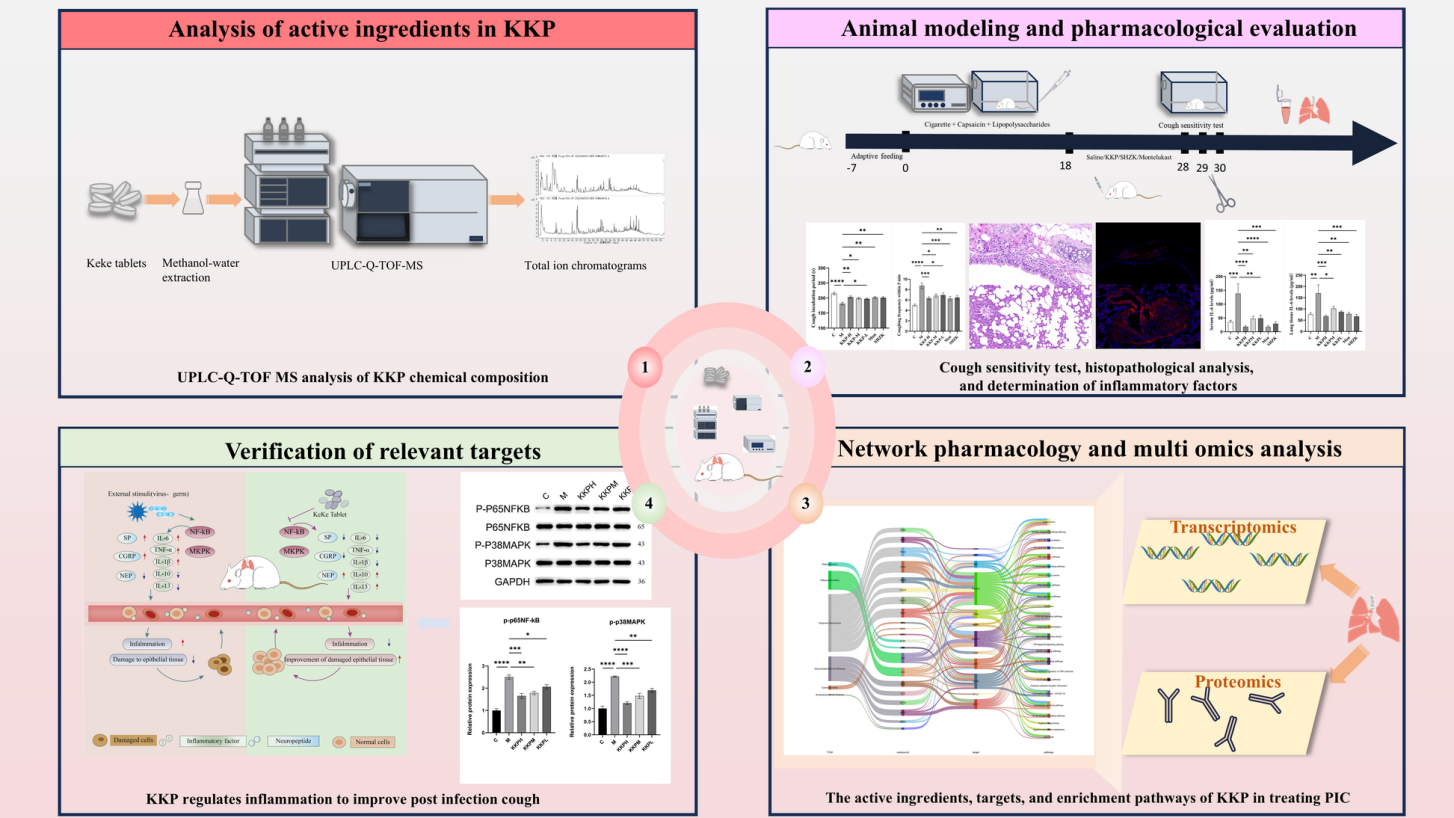
Schematic representation of the experimental design

This study aims to evaluate the therapeutic effects of Keke Tablets on PIC in rat models through behavioral assessments, pathological examinations, and molecular biology techniques. Additionally, molecular biology and multi-omics analyses will investigate the comprehensive effects of Keke Tablets in regulating PIC, while bioinformatics will identify potential targets and pathways (Fig. 1).

## Materials and methods

### UPLC‒Q-TOF–MS/MS analysis of KKP components

After crushing and screening the Keke tablets, a 0.5 g sample was placed into a conical flask. Subsequently, 25 mL of a 50% methanol solution was added, and the mixture was sonicated for 30 min. The flask was reweighed following the addition of 50% methanol water, shaken vigorously, and allowed to stand before the supernatant was filtered. UPLC-Q-TOF–MS/MS was utilized for the identification of chemical components in the methanol–water extract of Keke tablets. The extracts were separated on a Waters CORTECS T3 column and eluted with a gradient of acetonitrile(B) and 0.1% formic acid in water(A) (0.3 mL/min, 40 ℃). The total ion current map was generated by scanning in both positive and negative ion modes, using specific MS parameters including ion spray voltage, drying gas temperature, atomizing gas pressure, flow rates, electrospray voltage, and scanning range [70].

### Animal handing

Experimental Sprague Dawley (SD) rats (6 weeks, male, Weighing 200± 20 g) were obtained from Jinan Pengyue Experimental Animal Breeding Co., Ltd. They were housed at the Experimental Animal Center of Shandong University of Traditional Chinese Medicine under standardized conditions. We followed all guidelines set by the National Institutes of Health (NIH) for the care and use of laboratory animals in the U.S. and were approved by Shandong University of Traditional Chinese Medicine's Experimental Animal Ethics Review Board (Welfare Ethics License number: SDUTCM20230807001-1).

The rat model of post-infectious cough (PIC) was established by exposure to cigarette smoke, nebulization of capsaicin, and intranasal instillation of lipopolysaccharide (LPS) [83]. Except for the control group (n = 15), the remaining rats (n = 90) were exposed to a mixture of 50 g sawdust and 10 Yuxi cigarettes (Hongtashan, Yuxi, Yunnan) in a chamber (0.5 m^3^) for 30 min daily over 10 days. On the 11th, 14th, and 17th days, all rats except the control group received isoflurane (2.5%, Shenzhen Rwdls Life Technology Co., LTD) anesthesia, and a PBS solution containing LPS (0.4 mg/ml, with a calculated intranasal dose of 1 μL/g body weight, Sigma-Aldrich) was administered intranasally. Additionally, rats were exposed to CAP (10^−4^ M, Beijing Solarbio Science & Technology Co., LTD) aerosol in a transparent chamber once daily for 3 min on days 12, 13, 15, 16, and 18. After modeling, rats (excluding the control group) were randomly divided into six groups (n = 15 each): model group (M, normal saline), low-dose Keke Tablet (KKPL, 194.4 mg/kg), medium-dose Keke Tablet (KKPM, 388.4 mg/kg), high-dose Keke Tablet (KKPH, 777.6 mg/kg), montelukast group (Mon, 0.9 mg/kg), and Suhuang Zhike Capsule group (SHZK, 364.5 mg/kg) as a positive control. The drug dosage for rats was determined based on the equivalent dose conversion between Human and rat body surface area. Each group received the designated treatment by gavage for 10 days. 24 h after the last administration, each group of rats were placed in a cough inducing and asthma inducing device and nebulized with capsaicin solution for 3 min. Starting from the first cough, record the cough latency and the number of coughs within 5 min after nebulization. Afterwards, the rats were sampled. Firstly, the rats were anesthetized with isoflurane inhalation and then bled from abdominal aorta. the tracheal tissue, lung tissue, and serum of the rats were collected and stored in a −80 ℃ freezer for subsequent experimental analysis.

### Lung and trachea histopathology observation and evaluation

The left lung and tracheal tissues were excised from sacrificed rats and immediately immersed in 4% paraformaldehyde solution (Wuhan Servicebio Biotechnology Co., Ltd.) at room temperature for 24 h to ensure adequate fixation. After fixation, the tissues were rinsed three times with phosphate-buffered saline (PBS) to remove residual fixative. The tissues were then dehydrated through a graded ethanol series (70%, 80%, 95%, and 100%), cleared in xylene, embedded in paraffin wax, and sectioned into 4 μm-thick slices using a rotary microtome. Sections were placed on glass slides, baked at 60 °C for 1 h, dewaxed in xylene, and rehydrated through graded ethanol to distilled water.

For histopathological evaluation, sections were stained with hematoxylin for 5 min and eosin for 2 min (HE staining; Beijing Solarbio Science & Technology Co., Ltd.), dehydrated, cleared, and sealed with neutral resin. Images were captured using a light microscope (Nikon Eclipse E100), and tissue structure and pathological changes were evaluated.

For immunofluorescence staining, paraffin sections were dewaxed, rehydrated, and subjected to antigen retrieval by boiling in citrate buffer (pH 6.0) for 20 min using a microwave oven. After cooling to room temperature, sections were encircled with a hydrophobic barrier pen and treated with autofluorescence quenching reagent for 5 min (Wuhan Servicebio). Non-specific binding was blocked with 3% bovine serum albumin (BSA) in PBS for 30 min at room temperature.

The sections were incubated overnight at 4 °C with the following primary antibodies, diluted in antibody diluent: Substance P (SP,1:200, DF7522), calcitonin gene-related peptide (CGRP,1:200, DF7386), and neutral endopeptidase (NEP,1:200, DF7446) (all from Affinity Biosciences).

After primary incubation, the slides were rinsed three times with PBS and incubated with CY3-conjugated goat anti-mouse secondary antibody (Wuhan Servicebio) for 50 min at room temperature in the dark. Nuclear counterstaining was performed using DAPI (10 μg/mL) for 10 min. After washing, the slides were mounted with antifade mounting medium and sealed. Fluorescent images were captured using a fluorescence microscope and analyzed with ImageJ software to quantify fluorescence intensity and localization.

### Cytokine analysis

Lung tissue homogenates and serum samples were collected from experimental rats. The levels of inflammatory cytokines, including TNF-α, IL-6, IL-1β, IL-10 and IL-13, were quantified using enzyme-linked immunosorbent assay (ELISA) kits (Hangzhou Lianke Biotechnology Co., Ltd. Hangzhou, China), following the manufacturer’s instructions. Absorbance was measured at 450 nm with a reference wavelength of 630 nm using a microplate reader. The concentrations of the cytokines were calculated based on standard curves generated from known concentrations of each analyte.

### Network pharmacological analysis

The components of KKP were initially retrieved from the HERB database (http://herb.ac.cn). The SMILES representations of these components were subsequently obtained from the PubChem database and imported into the ADMETlab 3.0 platform for pharmacokinetic evaluation. Active components were screened based on Lipinski's Rule of Five and ADME principles. The two-dimensional structures of these active components were then submitted to the SwissTargetPrediction web server, with the species parameter set to"Homo sapiens"while maintaining default settings for other parameters, to identify potential target sites. A network illustrating the relationship between active components of traditional Chinese medicine and their corresponding targets was constructed using Cytoscape software (version 3.9.1).

To identify disease-related targets,"Post-Infectious Cough"was used as a search term in both the GeneCards database (https://www.genecards.org/) and the OMIM database (http://www.omim.org/). Target genes obtained from these databases were consolidated, deduplicated, and standardized using the UniProt protein database. The intersection between KKP predicted targets and post-infectious cough-related targets was determined through Venn diagram analysis using the Weishengxin platform (https://www.bioinformatics.com.cn/), with the overlapping targets considered as potential therapeutic targets of KKP for post-infectious cough treatment. These intersection targets were subsequently incorporated into Cytoscape to construct a comprehensive drug-active ingredient-target interaction network.

For protein–protein interaction (PPI) analysis, the intersection targets were submitted to the STRING database with the species parameter set to"Homo sapiens"and a confidence score threshold of > 0.9. The resulting PPI network was visualized using Cytoscape software, from which free nodes were removed. Network topology analysis was performed using the CytoNCA plugin to identify key core targets.

Functional enrichment analysis was conducted using the Metascape platform (http://metascape.org/). The common targets of KKP and post-infectious cough were uploaded as a gene list, with"Homo sapiens"selected for both species input and analysis parameters. Custom analysis was performed, including Gene Ontology (GO) enrichment analysis (covering Molecular Functions, Biological Processes, and Cellular Components) and Kyoto Encyclopedia of Genes and Genomes (KEGG) pathway analysis. The results of these analyses were subsequently visualized using the Weishengxin platform.

### Transcriptomic analysis

Twenty-four hours after the final administration, rats in each group were anesthetized with isoflurane and sacrificed. Lung tissues were rapidly dissected on ice, placed into 5 mL cryogenic tubes, flash-frozen in liquid nitrogen, and stored at − 80 °C until further analysis. Rat lung tissue's total RNA was extracted by TRIzol. The mRNAs with a PolyA structure were enriched with oligo(dT) beads, fragmented, and reverse-transcribed into complementary DNA (cDNA). The purified cDNA underwent end-repair, A-tailing, and adapter Ligation. Selected cDNA fragments of 250–300 bp were PCR-amplified and purified to form a Library. The quality of the Libraries was assessed using the Agilent Bioanalyzer 2100, and those that passed quality checks underwent Illumina sequencing, yielding 150 bp paired-end reads. Raw FASTQ data were processed with custom Perl scripts. With the help of Hisat2 (v2.0.5), we aligned paired-end clean reads with the reference genome index. Differential expression between conditions was analyzed using DESeq2 (v1.16.1), identifying differentially expressed genes (DEGs) based on |log2FoldChange|> 1 and *P* < 0.05. Subsequently, GO and KEGG enrichment analyses were conducted using the clusterProfiler R software to reveal the functional roles and pathways of these DEGs.

### DIA proteomics

Samples were pretreated in PCT tubes using the Barocycler high-pressure sample system (Westlake Omi), desalted using SOLAμ solid-phase extraction well plates, and fractionated on an XBridge Peptide BEH C18 column. A data-dependent acquisition (DDA) library was constructed using a DIONEX μLtiMate 3000/Orbitrap Exploris 480 mass spectrometer. Subsequently, data-independent acquisition (DIA) was performed on a Vanquish Neo UHPLC/FAIMS Pro^™^/Orbitrap Exploris 480 mass spectrometer. DIA data analyzed with DIA-NN v1.8.1 (version 1.8.1), leveraging a pre-built DDA spectral library. Peptides and proteins identified with < 1% FDR threshold. The data analysis included quality control, differential analysis, and functional analysis using statistical software (e.g., PCA, t-SNE, UMAP, and R language packages, R version 4.0). Differentially expressed proteins (DEPs) identified based on FC (≥ 1.2 or ≤ 0.83) and *P* < 0.05. Functional enrichment via GO database, KEGG database and UniProtKB database (https://www.uniprot.org). Utilizing the STRING database (https://string-db.org), the protein–protein interaction (PPI) networks were visualized with Cytoscape.

### Western blot assay

The lung tissue was lysed in RIPA buffer (G2002, Servicebio, Wuhan, China) containing PMSF (ST506-2, Beyotime, Shanghai, China). Supernatant was collected via centrifugation (13,000 rpm, 15 mis). Its protein content was measured using a BCA assay (CW0014, CWBIO, China). Lung tissue lysates were subjected to 8–20% SDS-PAGE for Western blotting and transferred to polyvinylidene difluoride membranes (PVDF, 1620177, BIO-RAD, USA). The protein expression levels of A2m (1:1000, AB316101, Abcam, UK), S100a8(1:1000, A15315, Abclonal, USA), Hoxa5(1:5000,AB140636, Abcam, UK), Tab2(1:2000,14410-1-AP, Proteintech, USA), Dab2ip (1:8000,23582-1-AP, Proteintech, USA), p-p65 NF-κB (1:1000,3033, CST, China), p65-NF-κB (1:500,10745-1-AP, Proteintech, USA), p-p38 MAPK (1:1000,AP0526, ABclonal, China), p38-MAPK (1:6000,14064-1-AP, Proteintech, USA), and GAPDH (10494-1-AP, Proteintech, USA), were analyzed using corresponding primary antibodies. Incubate overnight at 4 ℃, then take it out and place it on a shaker at room temperature for 1 h the next day. HRP-conjugated secondary antibody (7074, CST, China) was incubated for 2 h. Protein bands were visualized using ECL chemiluminescent-science (E412-02, Vazyme, China) and the intensities of the bands were quantified using Image J software.

### Statistical analysis

The entire dataset was processed and evaluated utilizing GraphPad Prism 8 software, employing either one-way or two-way analysis of variance (ANOVA) as appropriate. The findings are displayed as the average value, along with its standard error of the mean (SEM). Image analysis was conducted utilizing ImageJ software. Statistical significance was verified at *P* < 0.05, signifying a meaningful difference between the data sets.

## Results

### Component analysis of KKP

**Fig. 2:**
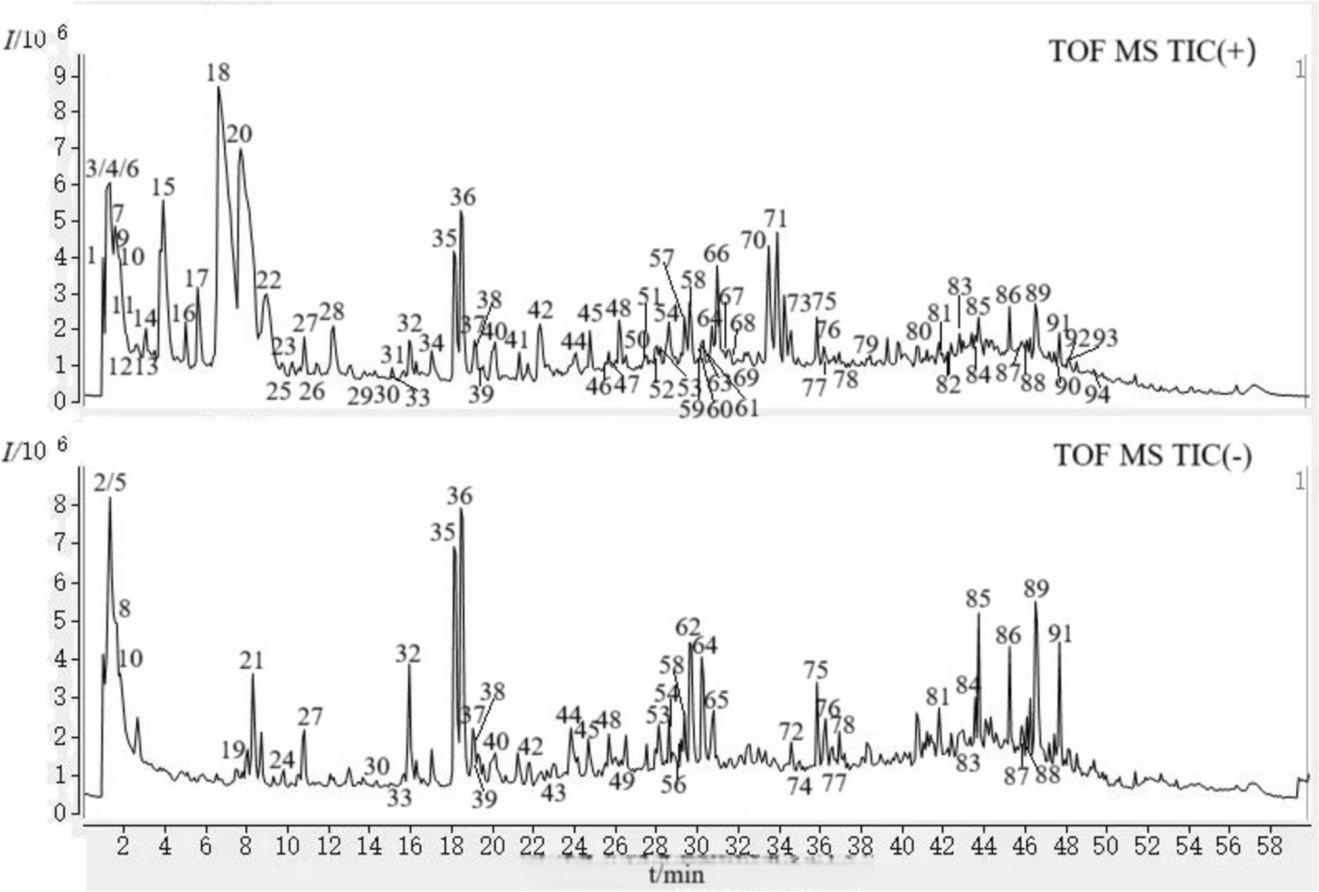
Total ion chromatograms for methanol-H_2_O extraction of KKP with UPLC-Q-TOF MS under positive mode and negative mode

To identify the active chemical components in the KKP sample, a comprehensive analysis was performed using UPLC-Q-TOF MS/MS. The total ion chromatograms obtained from the KKP sample in both positive and negative ion modes provided a detailed overview of its chemical composition (Fig. 2). The chromatographic peaks exhibited good separation, and the signals were characteristic. Precise Mass numbers for each peak were identified, leading to the identification of 94 compounds based on reference data, excimer ion peak mass numbers, secondary spectra, and relevant literature (Table S1). The identified compounds included 33 alkaloids, 22 flavonoids, 9 phenylpropanoids, 8 triterpene saponins, 5 amino acids, 3 cyanoglycosides, 6 organic acids, and 8 other compounds.

### KKP alleviates symptoms in PIC rats

During the modeling period, the control group of rats remained unaffected, displaying normal behavior, shiny fur, smooth breathing, and no abnormal signs. In contrast, rats in the model and treatment groups exhibited weight loss, dull fur, hair loss, increased irritability, shallow and rapid breathing, and wheezing. Following administration, the mental activity of rats in the KKP and positive control groups improved compared to the model group, their fur became shinier, and breathing stabilized.

**Fig. 3:**
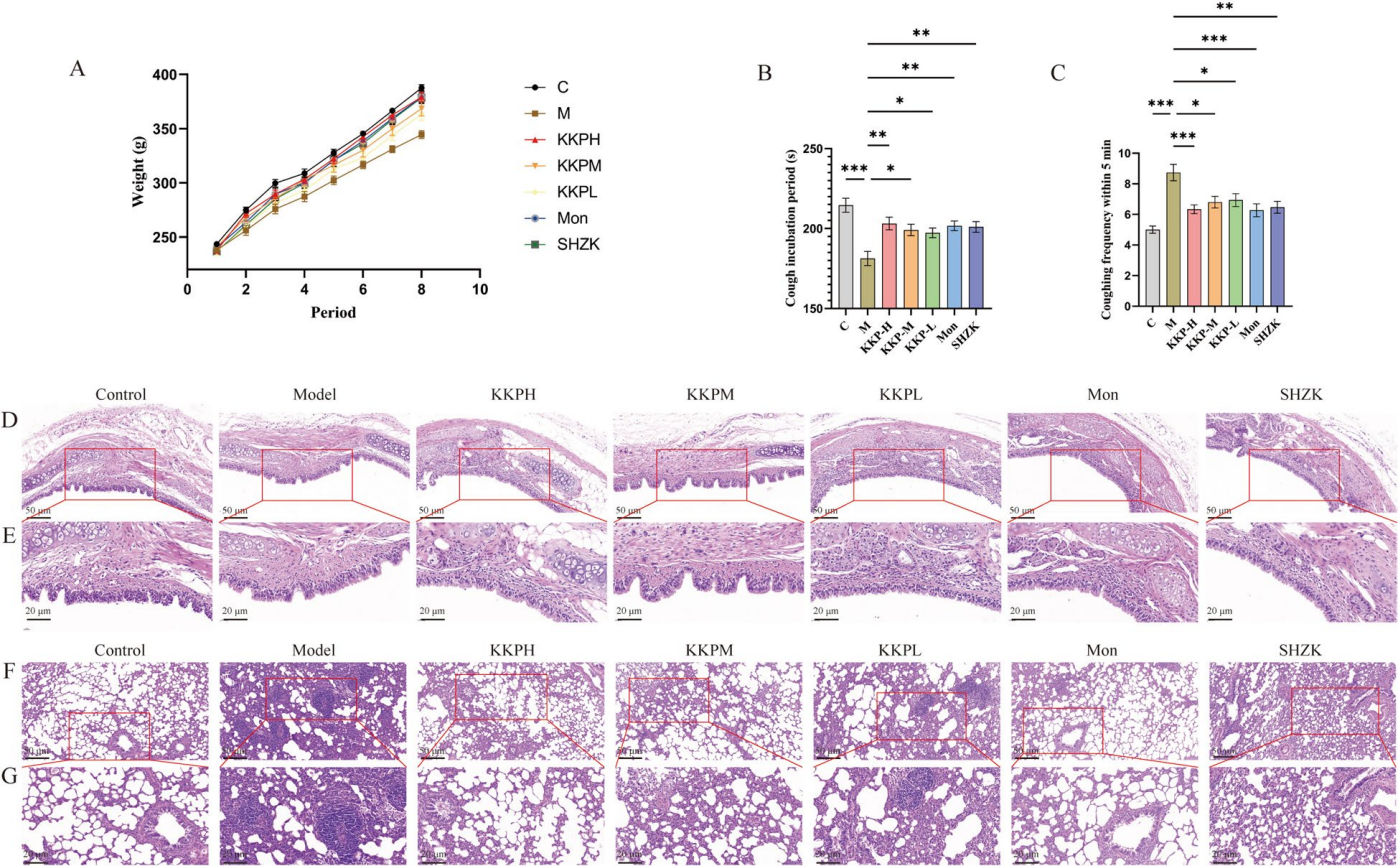
KKP showed a significant alleviating effect on PIC rats. **A** Changes in body mass of rats in each group (n = 15). **B** The incubation period of the first cough in each group of rats (n = 15). **C** Coughing frequency within five minutes in each group of rats (n = 15). **D**, **E** Representative images of H&E staining in the trachea of rats in each group (**D**: scale bar = 50 µm; **E**: scale bar = 20 µm). **F**, **G** Representative images of H&E staining in the lung of rats in each group (**F**: scale bar = 100 µm; **G**: scale bar = 50 µm). Data expressed as mean ± SEM; ***P < 0.001, **P < 0.01, *P < 0.05

As shown in Fig. 3A and Table 1, the body weight of rats in the control group continued to increase throughout the modeling period, while weight gain in the other groups was slower, with statistically significant differences. During the administration period, the body weight of rats in the model group recovered slowly, whereas those in the treatment groups gradually returned to near-normal levels, which was also statistically significant.

**Table 1:** Display of significant differences in body weight among different groups of rats

Period (measured every 5 days)	1	2	3	4	5	6	7	8
C vs M	NS	*	**	**	***	***	***	***
M vs KKPH	NS	NS	NS	NS	*	***	***	***
M vs KKPM	NS	NS	NS	NS	NS	NS	*	**
M vs KKPL	NS	NS	NS	NS	NS	NS	NS	*
M vs Mon	NS	NS	NS	NS	NS	**	***	***
M vs SHZK	NS	NS	NS	NS	*	*	***	***

^*^**P < 0.001; **P < 0.01; *P < 0.05; NS represents no significance

Cough sensitivity experiments (Fig. 3B, C) indicated that all groups exhibited varying degrees of coughing after capsaicin nebulization. The control group had longer cough latency and fewer coughs. In contrast, the model group's cough latency was significantly reduced, and cough frequency notably increased. Rats in the KKP and positive drug groups demonstrated prolonged cough latency and reduced cough frequency in a dose-dependent manner.

Histopathological observations from H&E staining (Fig. 3D, E) revealed disordered arrangement and inflammatory cell infiltration in the tracheal epithelial mucosa of the model group compared to normal rats. Damage to the trachea was improved in all KKP dose groups, as well as in the Montelukast and Suhuang Zhike Capsule groups, with high- and medium-dose KKP showing significant remission. In the model group, alveolar septa were widened, exhibiting angiogenesis, congestion, and inflammatory infiltration. The bronchioles showed protrusion and exfoliation into the lumen, with secretions present. Most alveolar structures in the Montelukast, Suhuang Zhike Capsule, and various KKP dose groups appeared normal, with some evidence of congestion and inflammatory infiltration, showing improvement compared to the model group (Fig. 3F, G). Notably, the high and medium dose KKP groups exhibited slightly better outcomes than the Montelukast, Suhuang Zhike Capsule, and low-dose KKP groups.

In summary, these results indicate that the PIC model was successfully established and that KKP demonstrates a significant protective effect in PIC rats.

### Overt anti-inflammatory activity of KKP in PIC models

**Fig. 4:**
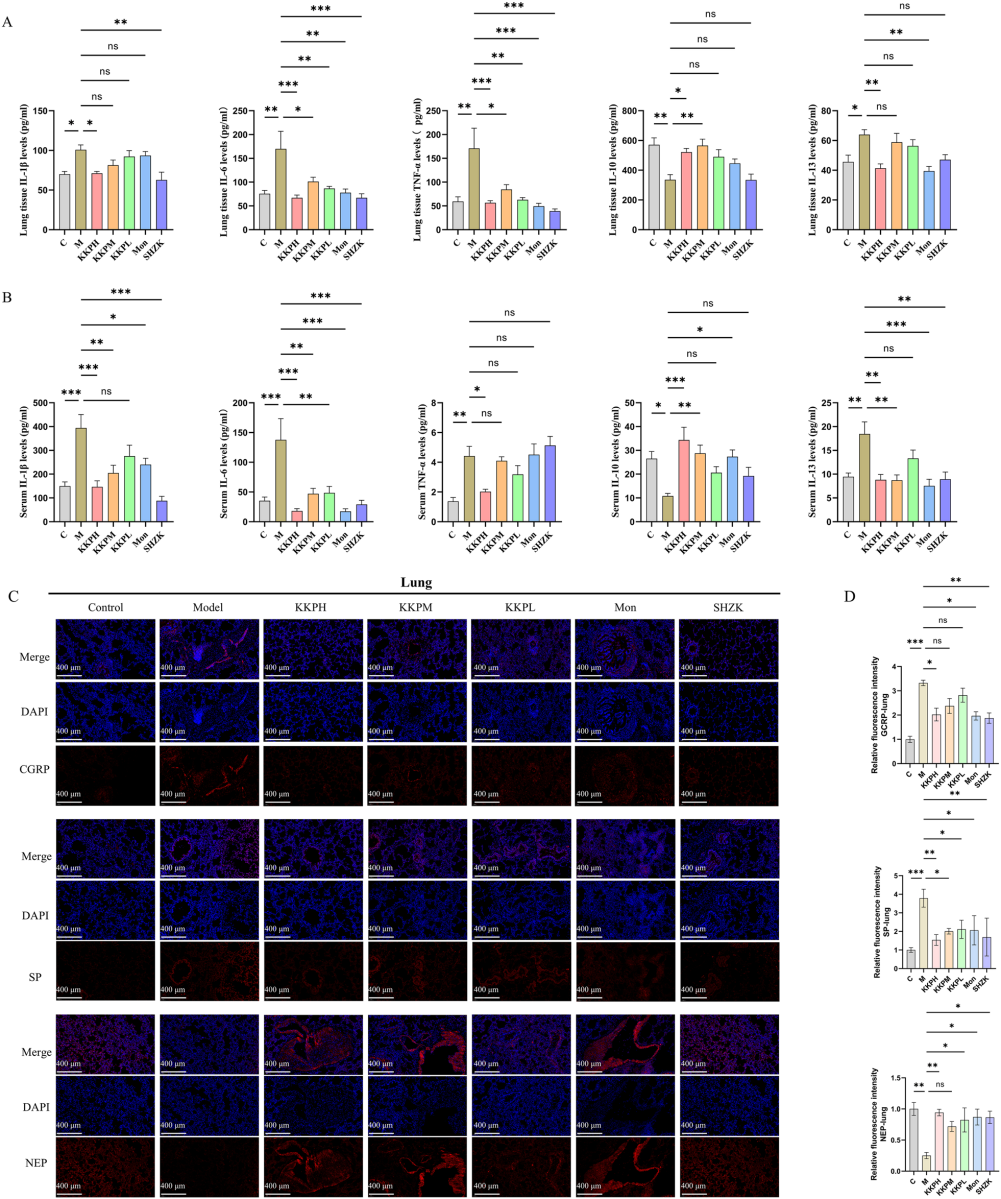

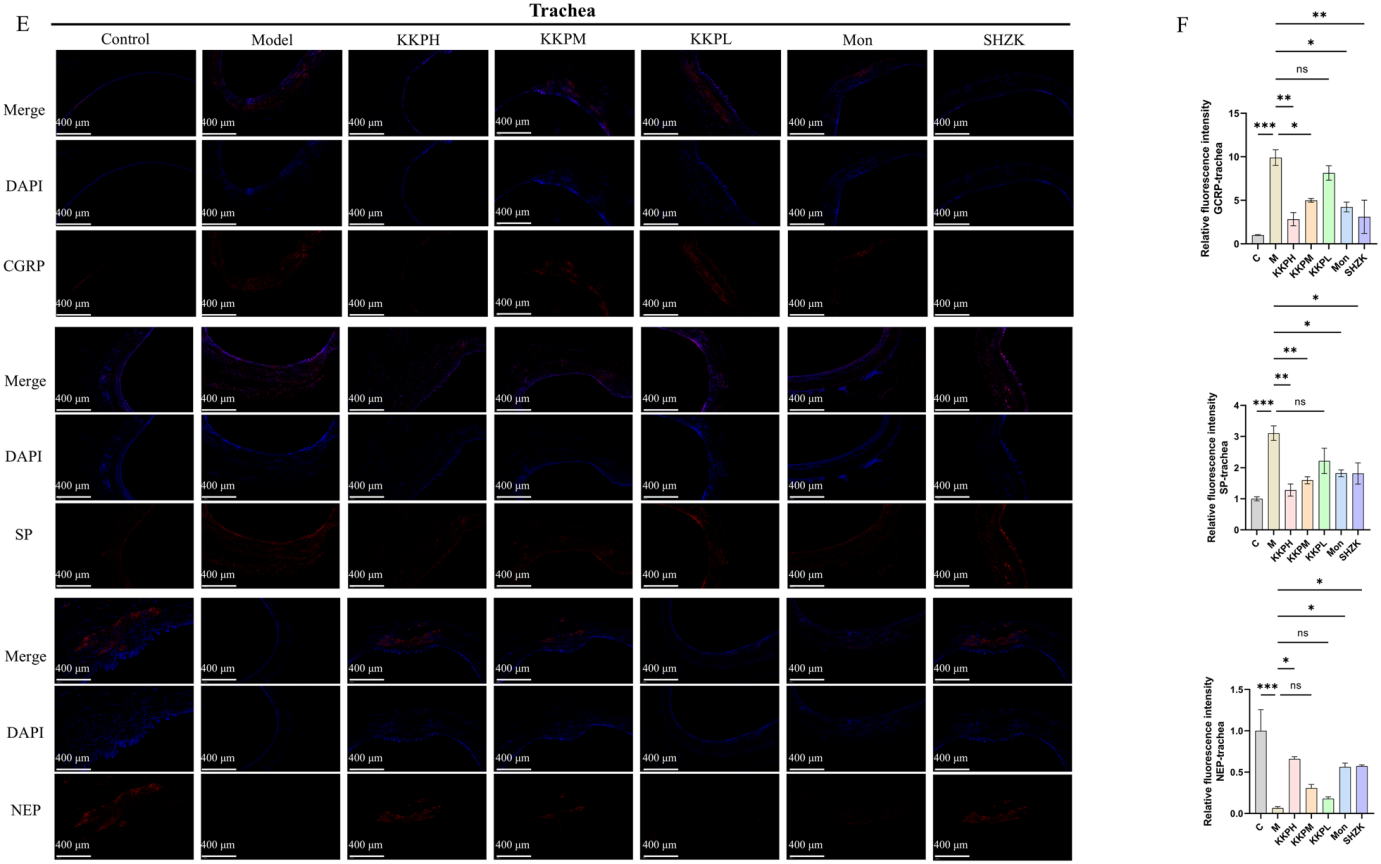
KKP administration alleviated inflammatory response in PIC rats. **A** The levels of inflammatory factors IL-1β, IL-6, TNF-α, IL-10 and IL-13 in lung tissue were measured by ELISA (n = 6). **B **The levels of inflammatory factors IL-1β, IL-6, TNF-α, IL-10 and IL-13 in serum were measured by ELISA (n = 6). **C** Representative images of immunofluorescence staining of lung in rats of each group (n = 4, scale bar = 400 µm). **D** Immunofluorescence results of lung tissue of each group. **E** Representative images of immunofluorescence staining of trachea in rats of each group (n = 4, scale bar = 400 µm). **F** Immunofluorescence results of trachea tissue of each group

Both serum and lung tissue were tested for cytokine levels to assess the anti-inflammatory effects of KKP in PIC. As shown in Fig. 4A, serum from PIC rats exhibited elevated levels of pro-inflammatory cytokines (TNF-α, IL-1β, IL-6 and IL-13) and decreased secretion of anti-inflammatory cytokines (IL-10) compared to controls. In contrast, treatment with high, medium, and low doses of KKP, along with positive control groups, significantly reduced inflammation by decreasing the expression of IL-1β、IL-6 and IL-13 while increasing the levels of IL-10. Similar trends were observed in lung tissue (Fig. 4B). Notably, higher concentrations of KKP demonstrated greater efficacy in alleviating inflammation, indicating that KKP effectively mitigates PIC-induced inflammatory responses.

Immunofluorescence results (Fig. 4C, D) revealed a marked increase in SP and CGRP fluorescence in the lungs of PIC rats compared to normal rats. Notably, subsequent treatment with KKP led to a significant attenuation of the SP and CGRP fluorescent signals. Additionally, KKP markedly enhanced the expression of NEP, as evidenced by the intensified NEP fluorescence. In tracheal tissue (Fig. 4E, F), KKP treatment resulted in notable improvements in neuroinflammation, characterized by increased NEP expression alongside decreased levels of SP and CGRP.

Collectively, these findings underscore the protective potential of KKP in mitigating neurogenic inflammation associated with the progression of PIC.

### Network pharmacology analysis

**Fig. 5:**
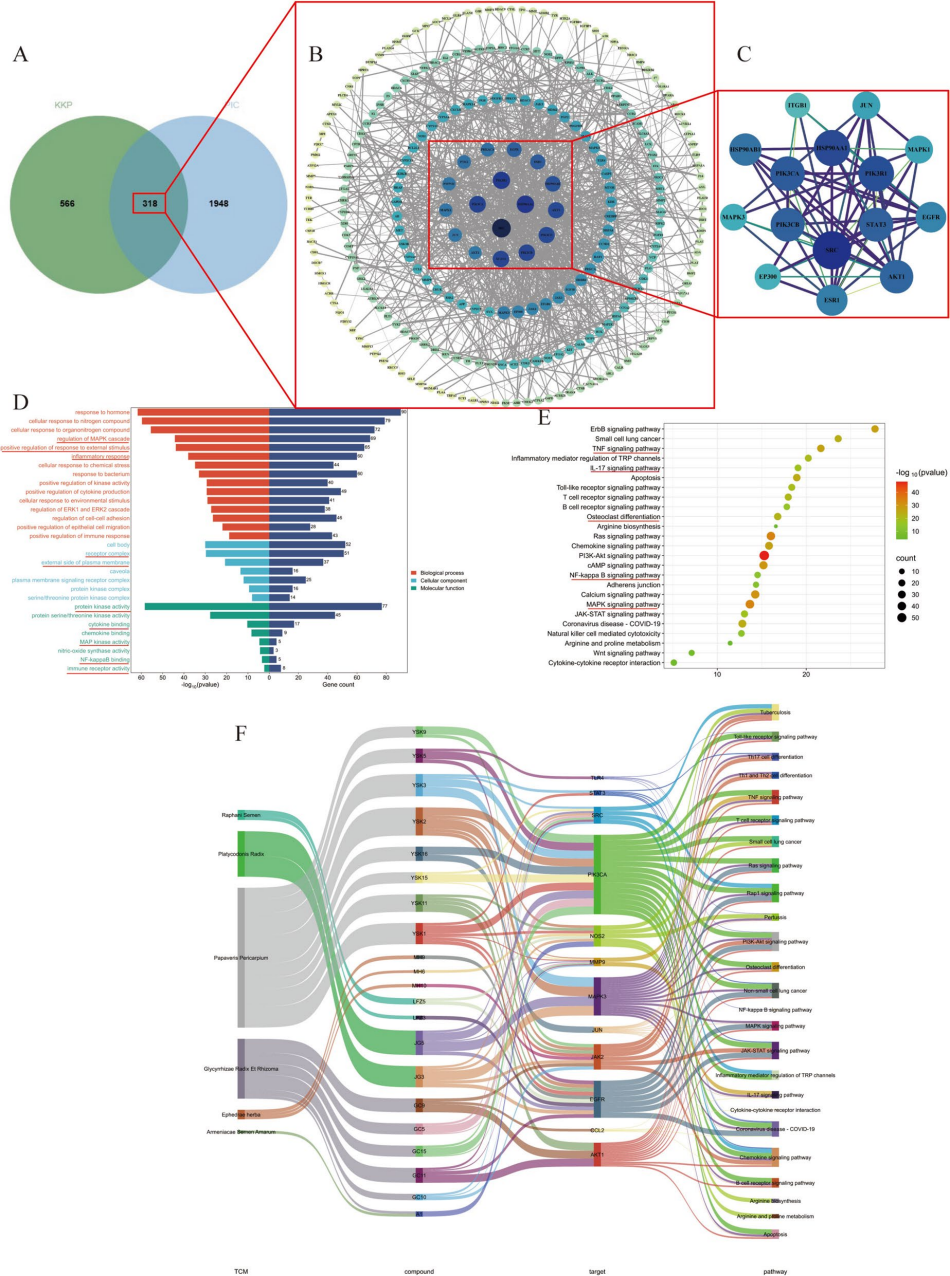
Network pharmacology analysis of KKP and PIC. **A** Venn diagram showing the overlapping targets of KKP and PIC. **B** Visualization of the PPI network using Cytoscape. **C** PPI network for the fifteen core genes. **D** GO analysis for the overlapping targets of KKP and PIC. **E** KEGG enrichment analysis for the overlapping targets of KKP and PIC. **F** TCM-compound-target-pathway network

A total of 1,569 chemical constituents were identified through a comprehensive Literature review and analysis using the Herb database. These included 492 from Ephedra, 180 from bitter almond, 600 from Glycyrrhiza, 151 from Platycodon grandiflorus, 87 from radish seed, and 58 from poppy shell. The composition of Gypsum was primarily obtained from literature sources, consisting mainly of calcium sulfate dihydrate (CaSO₄·2H₂O). Using SwissTargetPrediction 3.0, a total of 96 potential active compounds were identified, including 34 from Ephedra, 10 from bitter almond, 19 from Licorice, 7 from Platycodon grandiflorus, 6 from radish seed, 19 from poppy shell, and 1 from Gypsum. These active compounds corresponded to a total of 884 predicted target proteins. To explore the potential therapeutic mechanisms of Keke Tablets (KKP) in the treatment of post-infectious cough (PIC), disease-associated target proteins were retrieved from the GeneCards and OMIM databases using"post-infectious cough"as the keyword, and duplicate entries were removed. A total of 2,266 PIC-related targets were identified. Subsequently, the active ingredient targets of KKP were Mapped onto the PIC target set using a Venn diagram, revealing 318 overlapping genes, which were predicted as the key therapeutic targets of KKP against PIC (Fig. 5A).

The 318 shared targets were then analyzed through the STRING 12.0 database, with an interaction confidence score threshold set at 0.9, while isolated nodes were excluded. The resultant protein–protein interaction (PPI) network was visualized using Cytoscape 3.9.0. In this network, node size and color intensity corresponded to degree values (Fig. 5B). Core target proteins were Further screened based on network centrality using the CytoNCA plugin, revealing the hierarchical importance of targets within the PPI network. The top 15 core targets associated with KKP’s therapeutic effects on PIC included SRC, HSP90AA1, PIK3R1, PIK3CA, PIK3CB, STAT3, AKT1, HSP90AB1, ESR1, EGFR, MAPK1, JUN, MAPK3, EP300, and ITGB1 (Fig. 5C).

To further explore the biological functions of these targets, Gene Ontology (GO) functional enrichment and Kyoto Encyclopedia of Genes and Genomes (KEGG) pathway enrichment analyses were conducted using the Metascape database. GO analysis revealed that the significantly enriched biological processes (BP) were primarily related to the regulation of the MAPK cascade, positive regulation of responses to external stimuli, and inflammatory responses. The most enriched cellular components (CC) included receptor complexes and the external side of the plasma membrane. In terms of molecular function (MF), the key categories included protein kinase activity, cytokine binding, NF-κB binding, MAP kinase activity, and immune receptor activity (Fig. 5D). KEGG pathway analysis (Fig. 5E) highlighted key pathways associated with PIC pathogenesis and treatment, including the TNF signaling pathway, IL-17 signaling pathway, osteoclast differentiation, NF-κB signaling pathway, and MAPK signaling pathway. These findings suggest that inflammation-related pathways may play a crucial role in the therapeutic effects of KKP on PIC.

Finally, an integrated TCM-compound-target-pathway network for KKP was constructed based on the network pharmacology analysis. This network comprised 12 key targets, 21 prototype compounds, and 25 signaling pathways (Fig. 5F). The relationships among medicinal components, target proteins, and pathways in this network provide crucial insights into the underlying mechanisms of KKP in the treatment of post-infectious cough.

In conclusion, network pharmacology analysis revealed that KKP may alleviate symptoms of PIC by orchestrating multi-component, multi-target synergistic effects to modulate key inflammatory and immune signaling pathways, including NF-κB/MAPK, thereby attenuating inflammatory responses.

### KKP treatment altered the transcriptomic profile of PIC rat model

**Fig. 6:**
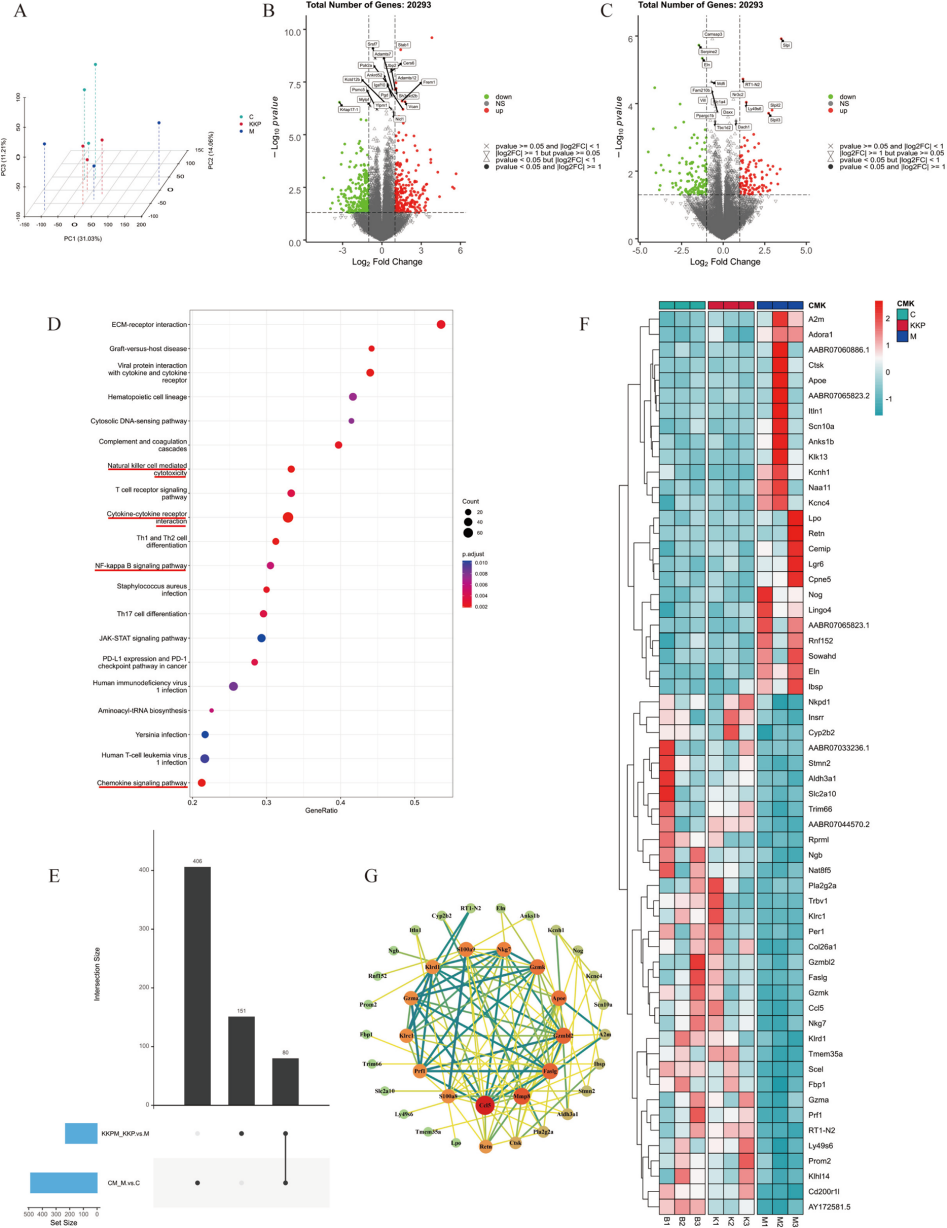
Transcriptomics identification for the mechanisms of KKP against PIC. **A** PCA for DEGs of Control, Model, and KKP group. **B** Volcano plot of DEGs between Control group and Model group. **C** Volcano plot of DEGs between Model group and KKP group. **D** GSEA pathway enrichment analysis for DEGs of KKP *vs.* Model. **E** Upset plot for DEGs of Control *vs.* Model, and Model vs. KKP. **F** Heatmap of the DEGs among Control, Model and KKP groups. **G** PPI Network Diagram of differential genes regulated by KKP

To further investigate the potential mechanism of the anti-infection cough effect of KKP, RNA-Seq was conducted. The PCA diagram (Fig. 6A) demonstrates a clear distinction between lung samples from PIC model rats and controls, highlighting significant differences in transcription profiles after model establishment. Notably, KKP-treated PIC rat lung samples clustered closely with controls, indicating that KKP substantially modulates lung gene expression in PIC rats. Volcano plots illustrated the overall distribution of DEGs. In comparison to the control group, a substantial alteration was noticed in the model group, with 486 gene sets significantly altered, comprising 242 up-regulated and 244 down-regulated genes (Fig. 6B). When comparing the KKP group to the model group, 231 DEGs were identified, with 107 genes up-regulated and 124 genes down-regulated (Fig. 6C). Additionally, hierarchical clustering heatmaps (Fig.S1A and Fig.S1B) showcased the top 20 most significant DEGs in each comparison, providing insight into gene expression patterns.

Subsequently, to gain insights into the functional roles of the identified DEGs, we analyzed GO and KEGG pathway analyses. The outcomes of the GO annotation analysis, comparing the control group with the model group, are presented in Fig.S1C. In terms of the BP the DEGs were predominantly enriched in categories such as"cell adhesion","cellular calcium ion homeostasis", and"positive regulation of natural killer cell-mediated cytotoxicity". In terms of MF, significant enrichment was noted for"signaling receptor binding,""transmembrane signaling receptor activity,"and"calcium-dependent protein binding."The CC analysis identified notable enrichment in"external side of plasma membrane,""extracellular matrix,"and"myofibril."Furthermore, the comparison between the model and KKP groups (Fig.S1D) highlighted enrichment in categories such as"neuronal action potential,""defense response,""positive regulation of natural killer cell-mediated cytotoxicity,""voltage-gated sodium channel activity,"and"extracellular matrix."The KEGG pathway analysis of the DEGs from the control vs. model group comparison revealed significant enrichment in pathways that are closely associated with inflammation and immunity, including"Cytokine-cytokine receptor interaction,""Natural killer cell mediated cytotoxicity,"and"Apoptosis"(Figure S1E). Similarly, the KEGG analysis of the DEGs from the model vs. KKP group comparison also highlighted the enrichment of pathways related to inflammation and immunity (Figure S1F). These findings underscore the potential involvement of these pathways in the underlying biological processes being studied, particularly in relation to inflammatory and immune responses.

To eliminate the gene enrichment analysis which depends on significant up or down regulation in the conventional pathway enrichment analysis, this study also used the GSEA pathway enrichment analysis method to analyze the expressed genes of all samples at the overall level. Genes regulated by KKP in the PIC model were significantly enriched in pathways such as"Natural killer cell-mediated cytotoxicity,""Chemokine signaling pathway,""NF-κB signaling pathway,"and"Cytokine-cytokine receptor interaction"(Fig. 6D). This is consistent with the KEGG findings, reinforcing the significance of these pathways in KKP's regulatory effects.

The result mentioned earlier a substantial overlap between the DEGs detected through the two comparative transcriptome analyses. Statistical analysis was conducted on these genes, revealing that 80 DEGs were shared between the two comparisons (Fig. 6E). To further explore changes in the relative content of these DEGs, a heatmap was generated (Fig. 6F), showing that mRNA expression levels in lung tissue of KKP-treated PIC rats resembled those of the control group, indicating that KKP treatment significantly influences mRNA expression. The PPI analysis of the DEGs was conducted, and a PPI network diagram was produced to further investigate the interaction and internal correlation among the translated proteins of the differentially expressed genes in the PIC rats treated with KKP. As shown in Fig. 5G, proteins such as Ccl5, Mmp8, Faslg, Gzmbl2, S100a9, Klrd1, and A2m exhibited high degree scores, suggesting they are closely associated with other proteins and may serve as key targets for KKP in regulating lung injury in PIC.

In conclusion, these transcriptomic results suggest that KKP may exert therapeutic effects on PIC rats through anti-inflammatory and immunomodulatory mechanisms.

### KKP treatment altered the proteomic profile of PIC rat model

**Fig. 7:**
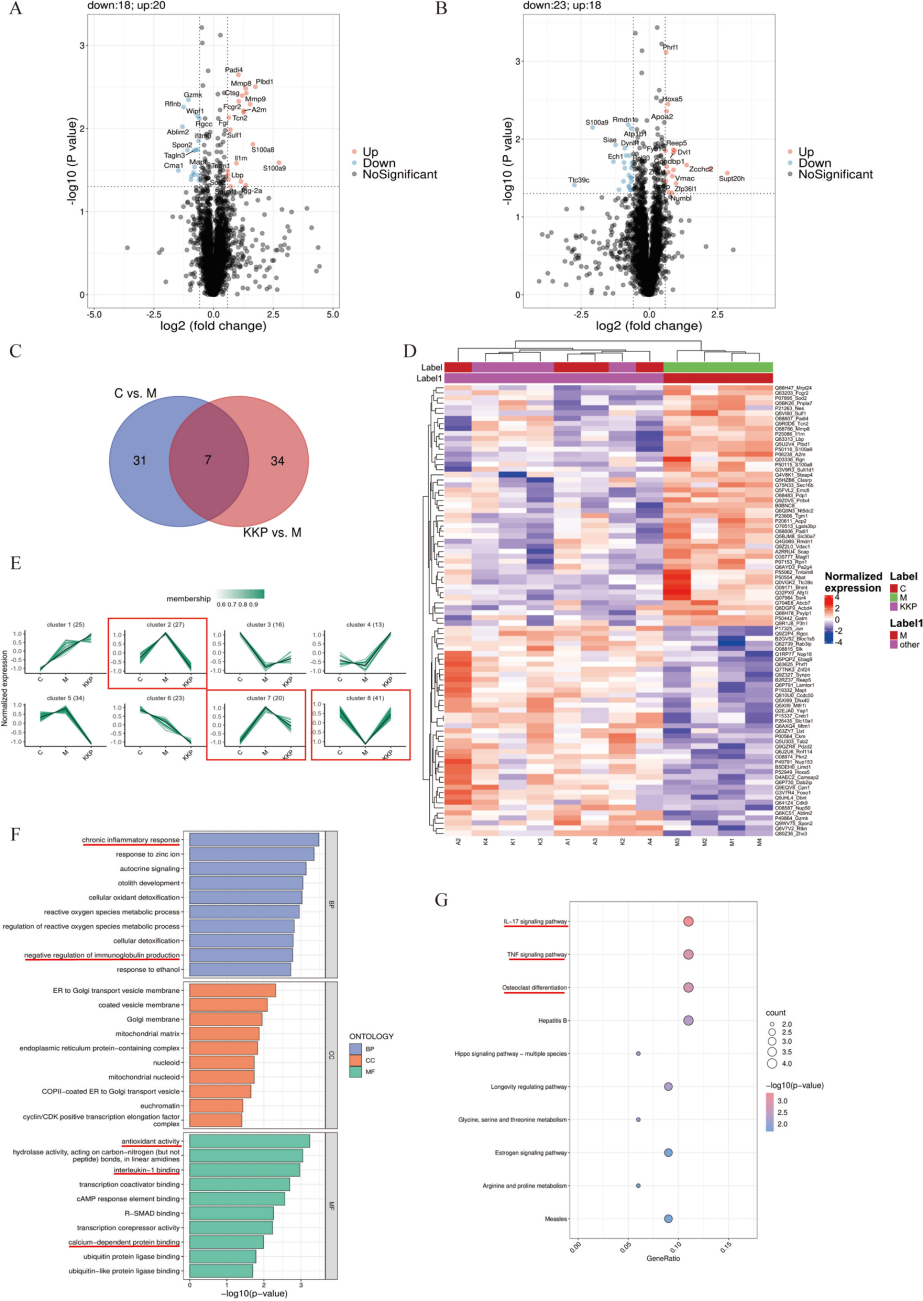
Proteomics identification for the mechanisms of KKP against PIC. **A** Volcano plot of DEPs between Control and Model group. **B** Volcano plot of DEPs between KKP and Model group. **C** Venn diagram exhibiting the distribution of overlapped DEPs. **D** Cluster heatmap of significantly different proteins in Control, Model, and KKP groups. **E** Cluster analysis of significantly changed proteins in Control, Model, and KKP groups based on their change tendencies. **F** GO enrichment analysis for DEPs of Control, Model and KKP groups. **G** KEGG enrichment analysis for DEPs of Control, Model and KKP groups

To Further investigate the mechanism of KKP intervention in PIC rats, we conducted DIA-based proteomic analyses on lung tissues from control, model, and KKP groups. A total of 31,214 peptides and 4,354 quantifiable, high-quality proteins were identified (Fig.S2A). Our analysis revealed 38 differentially expressed proteins (DEPs) in the comparison between the control and model groups, with 20 proteins upregulated and 18 downregulated (Fig. 7A). In the KKP versus model group comparison, we identified 41 DEPs, with 18 proteins showing up-regulation and 23 proteins exhibiting down-regulation (Fig. 7B).

To visualize the differential proteins between the control vs. model group and KKP vs. model group, Venn diagrams were created. As shown in Fig. 7C, seven DEPs were identified in the KKP group, including four downregulated and three upregulated proteins. Specifically, the expression levels of Limid, Hoxa5, Phrf1, and Ablim2 decreased in the model group but increased following KKP treatment. Conversely, A2m, S100a9, and Plbd1 proteins were upregulated in the model group but downregulated after KKP intervention.

Subsequently, GO and KEGG enrichment were performed based on all DEPs. For GO enrichment (Fig.S2B and S2C), In terms of BP, the DEPs in control vs. model group were enriched in"humoral immune response","Chronic inflammatory response"and"regulation of inflammatory response", while that in KKP group vs. model group were enriched in"negative regulation of immune effector process”. Referring to CC,"extracellular matrix"was the main location where DEPs existed in control vs. model group, while"cytosolic large ribosomal subunt","polysomal ribosome"were the DEPs located components in KKP group vs. model group. Regarding to MF, the DEPs in control vs. model group were enriched in"interleukin-1 binding","antioxidant activity","Calcium-dependent protein binding", while that in KKP group vs. model group were enriched in structural constituent of"calcium-dependent protein binding”. The above results show that the DEPs in control vs. model group were mainly related to immunity, inflammation, oxidation and calcium ion binding, while that in KKP group vs. model group also were enriched in immune regulation and calcium ion binding. In addition, for KEGG pathway enrichment (Fig.S2D and S2E), DEPs in control vs. model group were principally enriched in IL-17 signaling Pathway, while that in KKP group vs Model group were enriched in ribosome、coronavirus Disease-COVID-19.

We further analyzed the expression trends of proteins across the"control vs. model vs. KKP"groups and performed cluster analysis on the differential proteins (Fig. 7D). Proteins were grouped into eight clusters based on their change trends (Fig. 7E). Clusters 2 and 7 contained proteins that increased in the PIC rats but decreased with KKP treatment, while Cluster 8 included proteins that decreased in the PIC rats but increased with KKP intervention. Clusters 1, 3, 4, 5, and 6 showed no significant changes due to KKP. Proteins in Clusters 2, 7, and 8 were identified as KKP-regulated. For proteins fitting these trends, GO and KEGG pathway enrichment analyses were conducted again. GO enrichment (Fig. 7F) indicated significant associations with chronic inflammatory response, negative regulation of immunoglobulin production, antioxidant activity, interleukin-1 binding, and calcium-dependent protein binding. The KEGG pathway enrichment highlighted significant involvement in the osteoclast differentiation, TNF signaling pathway, and IL-17 signaling pathway (Fig. 7G), with common DEPs including S100a8, S100a9, Jun, Tab2, Creb1, and Dab2ip.

In conclusion, these proteomic results suggest that KKP may intervene in PIC by regulating inflammation-related signaling pathways.

### Analysis of transcriptomes and proteomes integrated

**Fig. 8:**
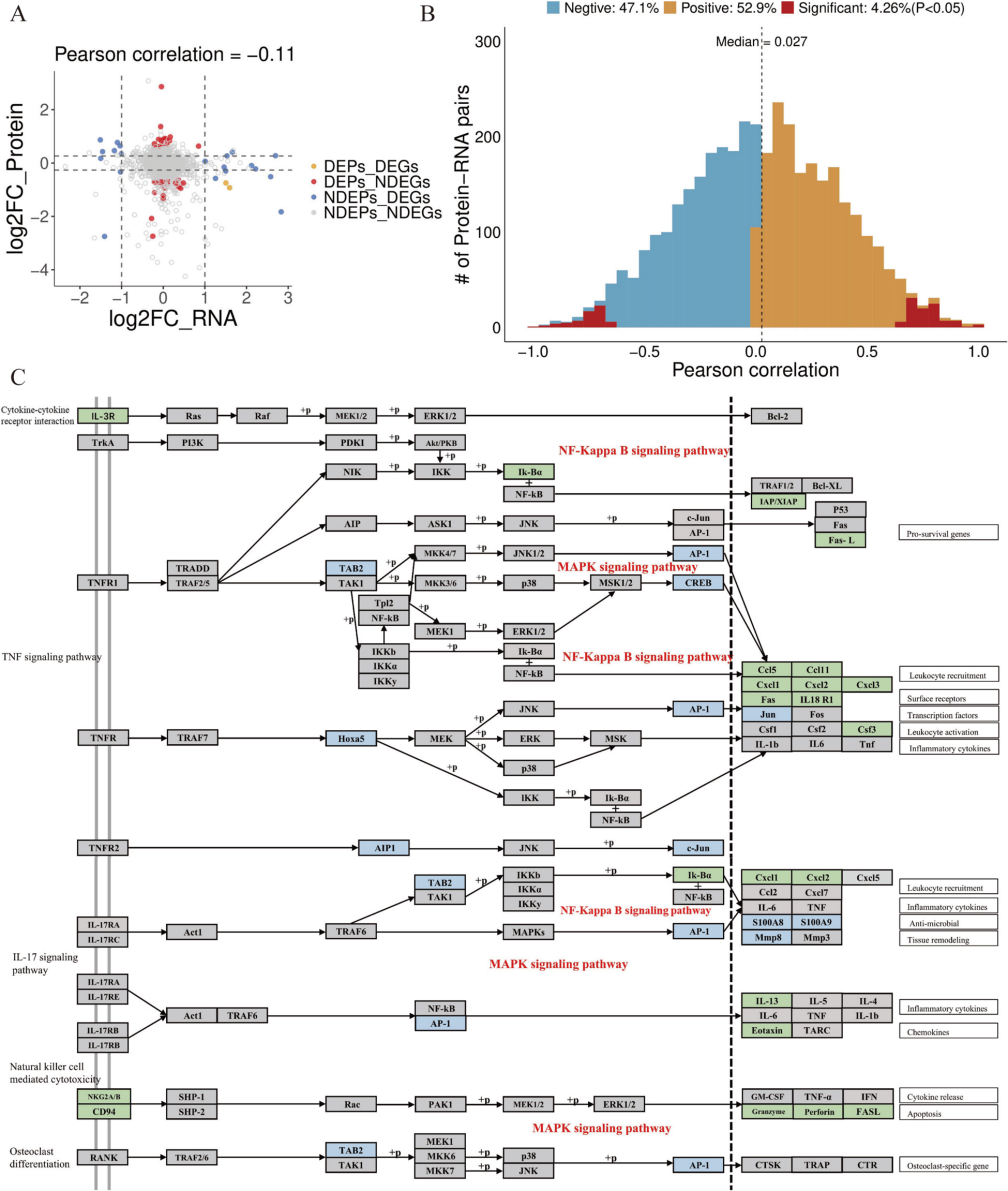
Integrated analysis of transcriptomics and proteomics. **A** The nine quadrants of DEG and DEP are processed by KKP. **B** Density plot showing the Spearman correlation coefficient r values between mRNAs and their corresponding proteins. **C** The specific pathway of KKP against PIC. The differentially expressed proteins in proteomic studies are labeled with blue, and the differentially expressed genes in transcriptional studies are labeled with green

To gain deeper insights into the specific mechanisms of KKP treatment for PIC, transcriptome and proteome data were analyzed comprehensively. The nine-quadrant diagram (Fig. 8A) illustrates the impact of KKP on PIC-related proteins and RNAs across nine quadrants. Quadrants 1, 2, and 4 showed lower RNA abundance compared to protein abundance, while quadrants 6, 8, and 9 indicated higher RNA abundance relative to proteins, potentially due to post-transcriptional or translational regulation. Quadrants 3 and 7 displayed a correspondence between RNA and associated proteins, whereas Quadrant 5 exhibited consistent expression of both without discernible differences. Correlation analysis yielded a coefficient of −0.11, indicating that most differentially expressed proteins did not correlate with their corresponding transcription levels. Our analysis identified 3,877 RNA–protein pairs in the two lung tissue groups, revealing a median correlation value of 0.027, with only 4.26% of RNA–protein pairs showing significant positive Spearman correlations (Fig. 8B). These results collectively suggest that KKP treatment of PIC may be regulated by post-transcriptional modifications.

Through an integrated analysis combining network pharmacology, transcriptomics, and proteomics, we have obtained a more comprehensive understanding of the molecular mechanisms underlying the therapeutic effects of KKP in the treatment of PIC. These three approaches revealed the intervention effects of KKP on PIC at multiple levels, with the results mutually corroborating one another and highlighting the regulation of inflammatory and immune signaling pathways. The therapeutic effect of KKP in the treatment of PIC may be accompanied by the regulation of NF-κB/MAPK signaling pathway. In our network pharmacology analysis, we identified 1,569 chemical constituents within KKP. Target prediction, utilizing SwissTargetPrediction and data mining from GeneCards and OMIM databases, revealed 318 targets associated with PIC. PPI network analysis, conducted via STRING and Cytoscape with the CytoNCA plugin, highlighted 15 core targets. Among these, key molecules such as MAPK1, MAPK3, JUN, STAT3, AKT1, and SRC are integral to inflammation and immune regulation pathways, including TNF, IL-17, NF-κB, and MAPK signaling. These results suggest that the active compounds in KKP may exert their anti-inflammatory and immunomodulatory effects by acting on key signal nodes such as the NF-κb/MAPK signaling pathway. Transcriptomic analysis further supports these conclusions. In lung tissues of PIC model rats, numerous DEGs were identified compared to controls. These DEGs are significantly enriched in pathways closely related to NF-κB and MAPK signaling, such as cytokine–cytokine receptor interactions, natural killer cell-mediated cytotoxicity, and apoptosis. GSEA analysis confirmed that genes modulated by KKP are significantly enriched in chemokine signaling and cytokine interaction pathways, underscoring the central role of inflammatory signaling regulation in KKP's therapeutic mechanism. The alignment of DEGs—including Klrc1, Klrd1, Prf1, Fasl, S100a8, and Mmp8—within these pathways suggests that KKP treatment restores dysregulated inflammatory responses in the PIC model, thereby attenuating pathological activation of NF-κB and MAPK pathways. Proteomic analyses corroborate these findings. Previous studies have demonstrated that KKP modulates proteins associated with the IL-17 signaling pathway, TNF signaling pathway, and osteoclast differentiation, all of which are linked to MAPK/NF-κB signaling. Proteomic profiling revealed differential expression of proteins such as TAB2, AP-1, CREB, Jun, Hoxa5, S100a8, S100a9, and Mmp8. In the PIC model, key inflammation-related proteins like A2m and S100a8 were upregulated, while proteins related to tissue repair and immune regulation, including Hoxa5, Tab2, and Dab2ip, were downregulated. Post-KKP intervention, these expression patterns were significantly reversed, indicating a close association between KKP treatment and the mitigation of the NF-κB/MAPK-mediated inflammatory cascade.

In summary, the multi-level data from integrated network pharmacology, transcriptomics and proteomics suggest that KKP reduces the production of pro-inflammatory factors and improves the inflammatory state of airway and lung tissue through the multi-components-multi-target synergistic action, accompanied by the regulation of inflammatory signaling pathways such as NF-κB and MAPK. In addition, KKP is able to regulate the expression of genes and proteins in the PIC model to a normal state, thereby alleviating the immune dysregulation and impaired lung function caused by PIC.

### Expression of potential pathway proteins was regulated by KKP

**Fig. 9:**
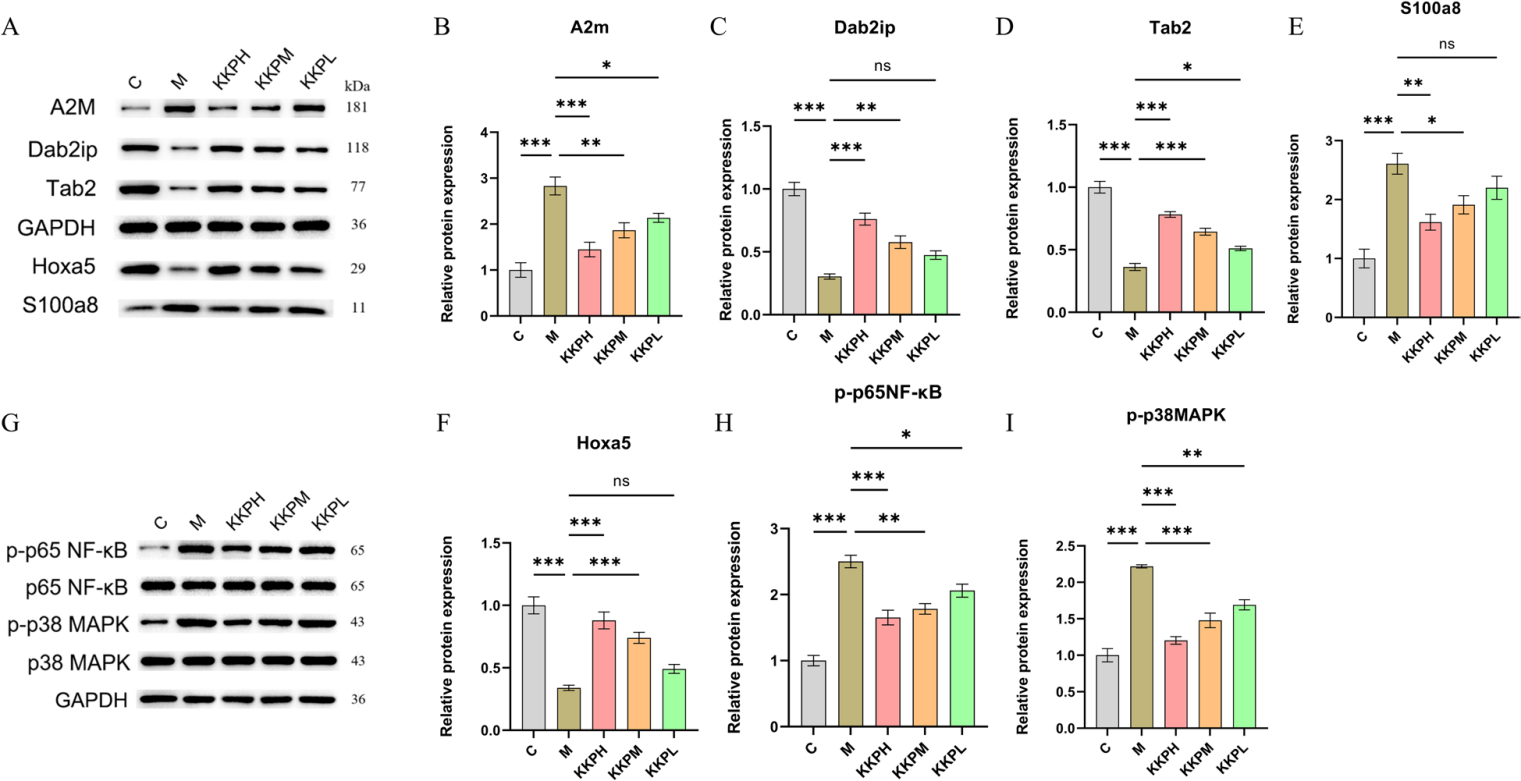
KKP regulates the expression of inflammation related pathway proteins. **A**, **B**, **C**, **D**, **E**, **F** The protein expression levels of A2m, Dab2ip, Tab2, Hoxa5, and S100a8 were detected using Western blot analysis (n = 6). **G**, **H**, **I** The protein expression levels of p-p65 NF-kB, p-65 NF-kB, p-p38MAPK and p-38 MAPK were detected using Western blot analysis (n = 3). Data expressed as mean ± SEM; ***P < 0.001, **P < 0.01, *P < 0.05, ns, not significant

To investigate whether KKP regulates the MAPK/NF-κB signaling pathways, we analyzed the expression of relevant proteins in the lung tissue of rats. As shown in Fig. 9, the model group exhibited significantly increased levels of A2m and S100a8 compared to the control group, while the expression of Hoxa5, Tab2, and Dab2ip was notably decreased. In contrast, KKP treatment effectively reduced the levels of A2m and S100a8 and enhanced the expression of Hoxa5, Tab2, and Dab2ip. Additionally, the levels of phosphorylated p38 MAPK (p-p38 MAPK) and phosphorylated p65 NF-κB (p-p65 NFκB) were elevated in the model group, but decreased following KKP intervention. These results further support that the beneficial effects of KKP can be mediated through the regulation of the MAPK/NF-κB signaling pathway.

## Discussion

A post-infectious cough (PIC) occurs after an acute respiratory infection and lasts for 3–8 weeks. It can significantly disrupt daily activities and sleep, severely affecting quality of life. Treatments typically include antihistamines, leukotriene receptor blockers, central antitussive drugs, and traditional Chinese medicine compounds, with combinations such as montelukast plus methoxyphenamine, or oxamide plus dextromethorphan showing enhanced efficacy [28, 35]. In this study, a rat model of post-infectious cough was established using a combination of cigarette smoking, aerosol inhalation of capsaicin, and nasal administration of lipopolysaccharide [83]. Researchers found that cigarettes increased cough sensitivity in the participant [2], while a 14-day exposure to cigarette smoke can establish a cough hypersensitivity model in guinea pigs [81]. Moreover, lipopolysaccharide (LPS) can induce cough hypersensitivity and lung inflammation in BALB/c mice [67]. Capsaicin is a potent activator of the TRPV1 channel residing on vagal C-fibers and is often used in research on cough sensitivity [11, 32]. In our investigation, the model group exhibited significant differences in body weight, cough latency, and cough frequency compared to the normal group, confirming the successful establishment of the PIC model in rats. Following KKP treatment, these conditions showed marked improvement.

The pathogenesis of PIC is complex and primarily involves heightened cough sensitivity in both peripheral and central nervous systems. External stimuli or infections lead to the recruitment of inflammatory cells, release of cytokines, and upregulation of TRP channels, resulting in neurogenic inflammation and airway nerve remodeling, which mediate peripheral nerve sensitization and ultimately increase cough sensitivity [39]. Cytokines such as TNF-α, IL-6, and IL-10, released during viral or bacterial infections, bind to specific receptors on C-fiber neurons of the vagus nerve, causing depolarization and increased excitability [14, 63]. Notably, TNF-α can enhance the sensitivity of lung sensory neurons, leading to increased cough sensitivity [46]. Furthermore, study has indicated that inflammatory factors can disrupt the tight connections of epithelial cell structures and modify the permeability characteristics of alveolar and airway epithelial cells, resulting in the infiltration and accumulation of activated inflammatory cells and following airway and lung tissue impair [55, 57]. Our findings revealed that KKP effectively suppressed key pro-inflammatory cytokines (TNF-α, IL-1β, IL-6) while promoting the expression of the anti-inflammatory cytokine IL-10 throughout the progression of PIC. The generation of inflammatory mediators, such as TNF-α and IL-6, correlates with the extent of lung tissue damage, underscoring their crucial role in the severity of histological lung injury [17, 78]. Histological analysis indicated that KKP significantly mitigated lung and tracheal damage caused by PIC, evidenced by reduced recruitment of inflammatory cells. These results suggest that KKP alleviates the inflammatory state in PIC rats while repairing damaged tissue and reducing cough sensitivity.

Upon exposure to viral, bacterial, and chemical stimuli, the TRP channel on the C fiber of the vagus nerve activates, upregulating TRPA1 and TRPV1 expression, enabling calcium ion influx [21, 80]. This depolarization triggers action potentials along the vagus nerve, releasing excitatory amino acids and stimulating neurons in the nucleus tractus solitarii and paratrigeminal nucleus, which project to the respiratory network, causing a reflexive cough [55]. Neuronal depolarization also causes sensory nerve endings (such as C-fiber endings) to release neuropeptides or transmitters such as SP, CGRP, etc. [61]. Simultaneously, airway epithelium damage caused by inflammation reduces neutral endopeptidase synthesis by epithelial cells and decreases neuropeptide degradation [16]. Neuropeptides and other substances act on effector cell receptors such as smooth muscle, cholinergic ganglion and blood vessel wall, causing bronchial smooth muscle contraction, capillary permeability increase, protein extravasation, mucous secretion increase, trachea mucosa congestion and edema and other neurogenic inflammation [4, 62]. Neuropeptides can also indirectly stimulate cough receptors, leading to a cough hypersensitivity state [25]. Immunofluorescence results showed decreased activity of NEP and elevated levels of SP and CGRP in the lungs of PIC rats, likely contributing to neurogenic inflammation. Notably, KKP administration resulted in increased NEP activity and decreased levels of SP and CGRP, indicating its potential to alleviate this inflammation. In summary, KKP have significant effects in the treatment of post-infectious cough. Through various mechanisms such as inhibiting inflammatory cell infiltration, reducing inflammatory cytokine secretion, and alleviating neurogenic inflammation, it can effectively diminish airway inflammation and airway hyperreactivity, thereby relieving cough symptoms.

Network pharmacology analysis was employed to elucidate the mechanism of action of KKP by integrating phytochemical constituents identified through experimental characterization and systematic database mining. Network pharmacology analysis revealed strong interactions among key target genes, including PIK3R1, PIK3CA, PIK3CB, STAT3, AKT1, MAPK1, JUN, MAPK3, HSP90, and TGB1. In studies on PIC, infections have been shown to alter immune responses, stimulate inflammatory reactions and cell proliferation, and subsequently induce cough symptoms. The genes PIK3CA, PIK3CB, and PIK3R1 regulate the proliferation and survival of immune cells through the PI3K/AKT pathway, playing a crucial role in cellular stress responses and immune regulation. These genes are likely to be involved in modulating inflammatory responses in infectious diseases. In particular, AKT1, as a downstream effector molecule, participates in the immune response to pathogens. Its abnormal activation can promote cytokine release, exacerbating both cough and inflammation [26, 58]. Additionally, MAPK1, MAPK3, and JUN play critical roles in inflammatory responses. Upon bacterial or viral infection, stimulation of the immune system activates the MAPK/ERK pathway, leading to cytokine and chemokine secretion, which exacerbates local inflammation and contributes to airway hypersensitivity and cough [31, 60]. Furthermore, STAT3 is also a key regulator in inflammatory responses, particularly in the early stages of airway inflammation, where its activation enhances immune cell reactivity, thereby worsening cough symptoms [18]. As a molecular chaperone, HSP90 (HSP90AA1 and HSP90AB1) is involved in cellular stress responses and protein stability regulation within the immune system. It plays a crucial role in post-infection tissue repair by stabilizing key transcription factors and signaling molecules such as STAT3 and AKT, thereby influencing immune and inflammatory responses, which in turn impact the onset and persistence of cough [69]. The ITGB1 gene, a key component of integrin signaling pathways, serves as a bridge in interactions between cells and the extracellular matrix, contributing to immune cell migration and positioning. Following infection, immune cells interact with airway epithelial cells via ITGB1, sustaining inflammation and exacerbating cough symptoms [22]. These genes are closely linked to post-infectious cough through their regulation of immune responses, cytokine secretion, and airway inflammation, suggesting that they may serve as important targets for KKP in alleviating PIC. Further KEGG pathway enrichment analysis of key targets demonstrated significant enrichment in pathways such as NF-κB and MAPK signaling, which are well-documented in the pathogenesis of PIC [51, 74]. NF-κB is a family of transcription factors whose activation generally leads to the production of pro-inflammatory cytokines, including TNF-α, IL-1, and IL-6, as well as chemokines and other immune mediators, thereby driving inflammatory and immune responses [5, 77]. Respiratory infections, particularly those caused by viruses (e.g., influenza virus, coronavirus) or bacteria, can activate the NF-κB pathway, triggering upper respiratory immune responses that promote airway inflammation and induce cough [3]. The MAPK signaling pathway, composed of multiple protein kinases. Different MAPK family members play distinct roles in cellular responses to stimuli. For example, JNK is closely associated with cellular stress responses and apoptosis, whereas p38 MAPK is crucial in inflammatory and stress responses [12, 68]. Viral infections can activate NK and p38 MAPK pathways, leading to the production of inflammatory mediators in epithelial cells, thereby promoting airway inflammation and triggering cough symptoms. Furthermore, the MAPK pathway is involved in airway smooth muscle proliferation and airway remodeling, processes that are critical in post-infectious cough and airway hyperresponsiveness [23, 50]. These studies collectively suggest that the abnormal activation of NF-κB and MAPK signaling pathways plays a pivotal role in the pathogenesis of PIC. The NF-κB pathway drives airway inflammation by promoting pro-inflammatory cytokine secretion, thereby exacerbating cough symptoms. Simultaneously, the MAPK pathway intensifies airway inflammation and cough by regulating cell proliferation, stress responses, and immune processes.

To investigate in greater detail the molecular processes involved in KKP intervention in PIC, we examined changes in mRNA and protein levels in lung tissue through transcriptomic and proteomic analyses. PCA of transcriptomics revealed that KKP treatment significantly shifts the transcriptional profile of PIC model rat lung tissues toward that of healthy controls, suggesting a systemic restoration of dysregulated gene expression [44]. Functional enrichment analysis indicated that KKP intervention regulated multiple genes within pathways related to inflammation and immunity, including"Cytokine-cytokine receptor interaction,""Natural killer cell mediated cytotoxicity,"and"IL-17 signaling pathway". NK cells play a crucial role in modulating inflammatory and immune responses through the secretion of cytokines and chemokines [1]. The IL-17 family is integral to host immune defense and the progression of inflammatory diseases, activating pathways such as NF-κB and MAPK, which promote cytokine release [54]. The chemokine signaling pathway is central to the immune system and closely linked to pathological processes such as inflammation, tumorigenesis, and progression [10]. Cytokines regulate immune cell functions and maintain immune balance while participating in inflammatory processes [38]. Cytokines and chemokines, such as TNF-α, upon binding to their respective receptors, can activate the p38MAPK and NF-κB signaling pathways, thereby participating in cellular inflammatory responses and apoptosis processes [64]. Additionally, these cytokines and chemokines could directly or indirectly activate airway sensory nerves, leading to cough hypersensitivity. Notably, the overlap of 80 DEGs between control vs. model and model vs. KKP comparisons points to a subset of genes directly influenced by KKP intervention. Among these, Ccl5, Mmp8, Faslg, and S100a9 emerged as high-degree nodes in the PPI network. CCL5 is a potent chemoattractant for immune cells and is upregulated in airway inflammation [53]. MMP8 contributes to tissue remodeling in chronic lung diseases [13], while S100A9, a calcium-binding protein, amplifies neutrophilic inflammation [37]. The reversal of these mediators by KKP aligns with its proposed anti-inflammatory effects.

Proteomics analysis showed that KKP treatment modified the expression of proteins involved in the IL-17, TNF signaling pathways, and osteoclast differentiation, such as Tab2, S100a8, Dab2ip, Jun, and Creb1. All three pathways mentioned above can mediate inflammatory signaling pathways such as NF-κB and MAPK through distinct receptors (including IL-17R, TNFR, and RANKL), subsequently inducing the expression and release of inflammatory factors, thereby regulating the progression of inflammatory responses [6, 41, 64]. A2M (Alpha-2-Macroglobulin) inhibits inflammatory cytokines, disrupting the inflammatory cascade [65]. According to research reports, the expression of A2M is decreased in the lung tissue and peripheral blood of patients with COPD (Chronic Obstructive Pulmonary Disease) [71]. In contrast, the expression of A2M is elevated in the joint tissue and blood samples of osteoarthritis model rats. After Taraxasterol treatment, which inhibits the NF-kB signaling pathway, the expression level of A2M approaches that of the control group [72]. Tab2 serves as a key regulator in the MAPK/NF-κB signaling cascade activated by inflammatory responses [76]. In various lung diseases, the S100a8/S100a9 complex exacerbates inflammatory responses by activating multiple cell surface receptors and intracellular signaling pathways (including MAPK and NF-κB), thereby promoting the emission of inflammatory mediators and the recruitment of immune cells [33, 59]. Dab2ip participates in multiple processes including natural immune response, inflammation, and cell growth [47]. The research suggests that Dab2ip mediates the balance between TNF/TRAF2-induced MAP3K5-JNK and NF-κB signaling pathways [79]. Hoxa5 plays a pivotal role in respiratory system development, as mutations can disrupt the formation of trachea, lung, and diaphragm, leading to respiratory failure and neonatal mortality [29, 36]. Our Western blot results showed that KKP treatment downregulated A2m and S100a8 while upregulating Hoxa5, Tab2, and Dab2ip, indicating its role in modulating immune, inflammatory, and morphogenetic pathways.

Subsequent combined analysis between network pharmacology, transcriptomics, and proteomics suggested that KKP may inhibit the expression of inflammatory molecules by regulating the MAPK/NF-κB signaling pathway, thereby reducing airway inflammation and reducing cough sensitivity. Previous studies have indicated that downregulating NGF, TrkA, and p-p38 MAPK expression can alleviate cough sensitivity. Furthermore, inflammation in animal models of post-infectious cough can be reduced by modulating the ERK signaling pathway, as well as the TLR4/MyD88/NF-κB and p38 MAPK pathways [19, 49, 73]. Based on these findings, we evaluated the effect of Keke Tablet on the MAPK/NF-κB pathway. Our data demonstrated that KKP effectively downregulated p65-NF-κB and p-p38 MAPK production in lung tissue, confirming its role in modulating these pathways.

## Conclusions

**Fig. 10:**
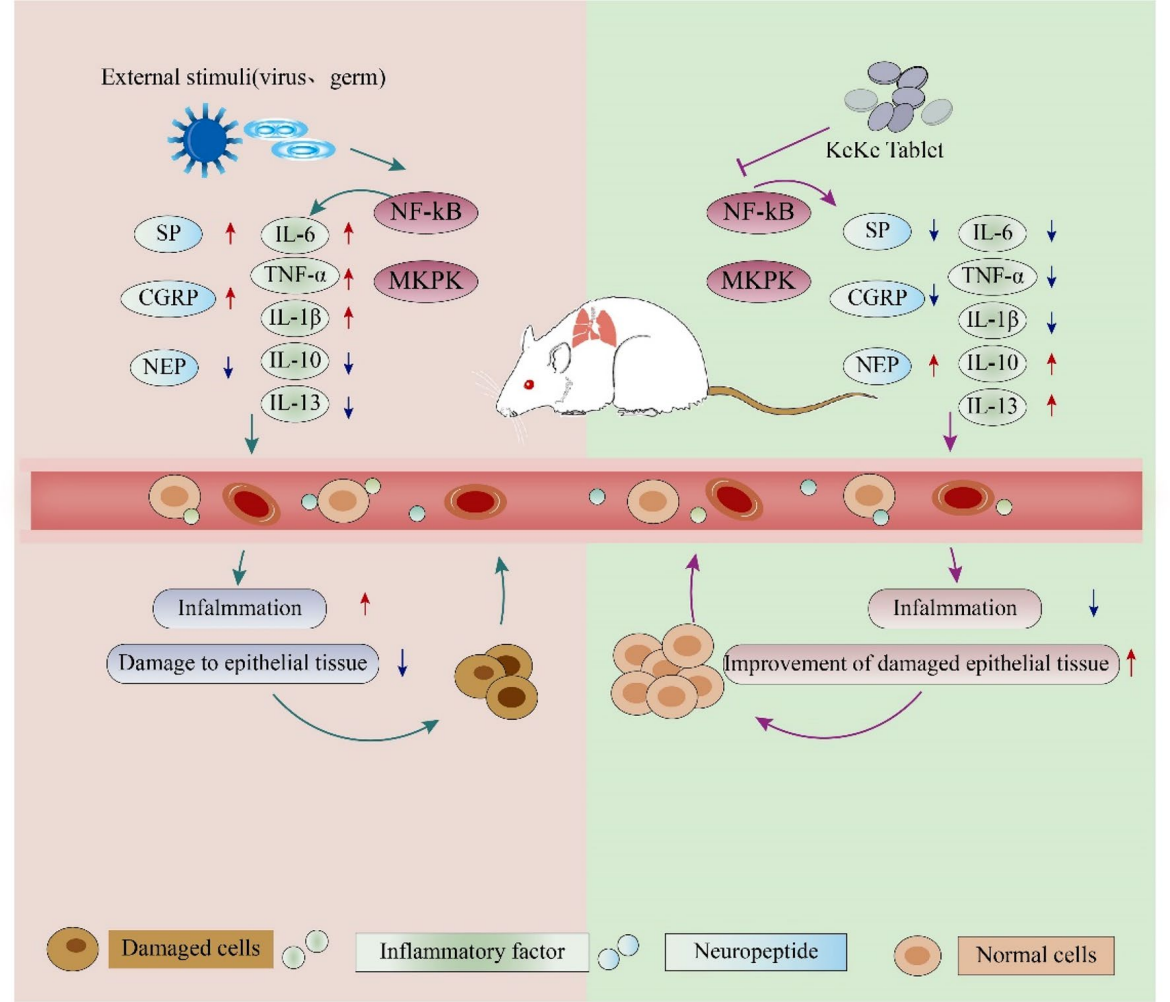
The potential role of KKP in PIC model rats. Multi-group studies have shown that KKP can regulate NF-kB/MAPK signal pathway, inhibit the expression of pro-inflammatory factors and neuropeptides, reduce the inflammatory response of airway and lung tissue, and restore damaged tissue

This study utilized an integrated approach involving network pharmacology, transcriptomics, proteomics, and molecular biology to validate the therapeutic effects of KKP on post-infectious cough. KKP can improve PIC mainly by repairing the damaged airway and lung epithelial barrier, reducing cough sensitivity, reducing inflammatory cytokine secretion and reducing inflammatory response. NF-κB/MAPK pathway is a potential pathway of Keke tablet in the treatment of post-infectious cough (Fig. 10). However, the detailed mechanisms of KKP and its components in PIC, as well as the specific active ingredients, require further investigation. Future research should focus on determining the bioavailability of KKP's primary components and exploring emerging technologies like metabolomics to elucidate the precise mechanisms of its active ingredients in blood. Ultimately, these efforts will enhance our understanding of the pharmacological mechanisms underlying KKP.

## Supplementary Information

Supplementary material 1.

## Data Availability

All data generated or analysed during this study are included in this published article.

## Abbreviations

PIC: Post-infectious cough
PBS: Phosphate buffer saline
UPLC-Q-TOF MS: Ultra-performance liquid chromatography-quadrupole tandem time-of-flight mass spectrometry
LPS: Lipopolysaccharide
CAP: Capsaicin
NEP: Neutral endopeptidase
SP: Substance P
CGRP: Calcitonin gene-related peptide
BSA: Bovine Serum Albumin
DAPI: 4’,6-Diamidino-2’-phenylindole
TNF-α: Tumor necrosis factor-α
IL-6: Interleukin-6
IL-1β: Interleukin-1β
IL-10: Interleukin-10
ELISA: Enzyme-linked immunosorbent assay
DEGs: The differentially expressed genes
DEPs: The differentially expressed proteins
GO: Gene Ontology
KEGG: Kyoto Encyclopedia of Genes and Genomes
PPI: Protein interaction network
FDR: False discovery rate
BP: Biological processes
MF: Molecular functions
CC: Cellular components
TRP: The transient receptor potential
TRPA1: Transient receptor potential ankyrin 1
TRPV1: Transient receptor potential vanilloid receptor 1
MAPK: Mitogen-activated protein kinase
