# Supplementary material for: Multi-omics and network pharmacology approaches reveal the mechanism of action of KeKe tablet against post-infectious cough
Source: Chin Med. 2025 Oct 4;20:160. doi: 10.1186/s13020-025-01199-7 (PMC12495694; doi:10.1186/s13020-025-01199-7)
Supplement: Supplementary file 1 — Supplementary material 1. [file 13020_2025_1199_MOESM1_ESM.docx]

Supporting information

Keke Tablet attenuates Inflammatory Responses via Modulation of MAPK/NF-κB Signaling Pathway in Post Infectious Cough: A Combined Multi-Omics and Molecular Biology Analysis

Figure Caption

**Fig.S1.** Transcriptomics identification for the mechanisms of KKP against PIC. (A) Top 20 DEPs compared between the Control and Model group in the heatmap. (B) Top 20 differentially expressed genes compared between the KKP and Model group in the heatmap. (C) GO enrichment analysis for DEGs of Control vs. Model. (D) GO enrichment analysis for DEGs of KKP vs. Model. (E) KEGG enrichment analysis for DEGs of Control vs. Model. (F) KEGG enrichment analysis for DEGs of KKP vs. Model.

**Fig.S2.** Proteomics identification for the mechanisms of KKP against PIC. (A) Statistical histogram of identification and quantitative results. (B) GO enrichment analysis for DEPs of Control vs. Model. (C) GO enrichment analysis for DEPs of KKP vs. Model. (D) KEGG enrichment analysis for DEPs of Control vs. Model. (E) KEGG enrichment analysis for DEPs of KKP vs. Model.

**Fig.S1.** (A) Top 20 DEPs compared between the Control and Model group in the heatmap. (B) Top 20 differentially expressed genes compared between the KKP and Model group in the heatmap. (C) GO enrichment analysis for DEGs of Control vs. Model. (D) GO enrichment analysis for DEGs of KKP vs. Model. (E) KEGG enrichment analysis for DEGs of Control vs. Model. (F) KEGG enrichment analysis for DEGs of KKP vs. Model.


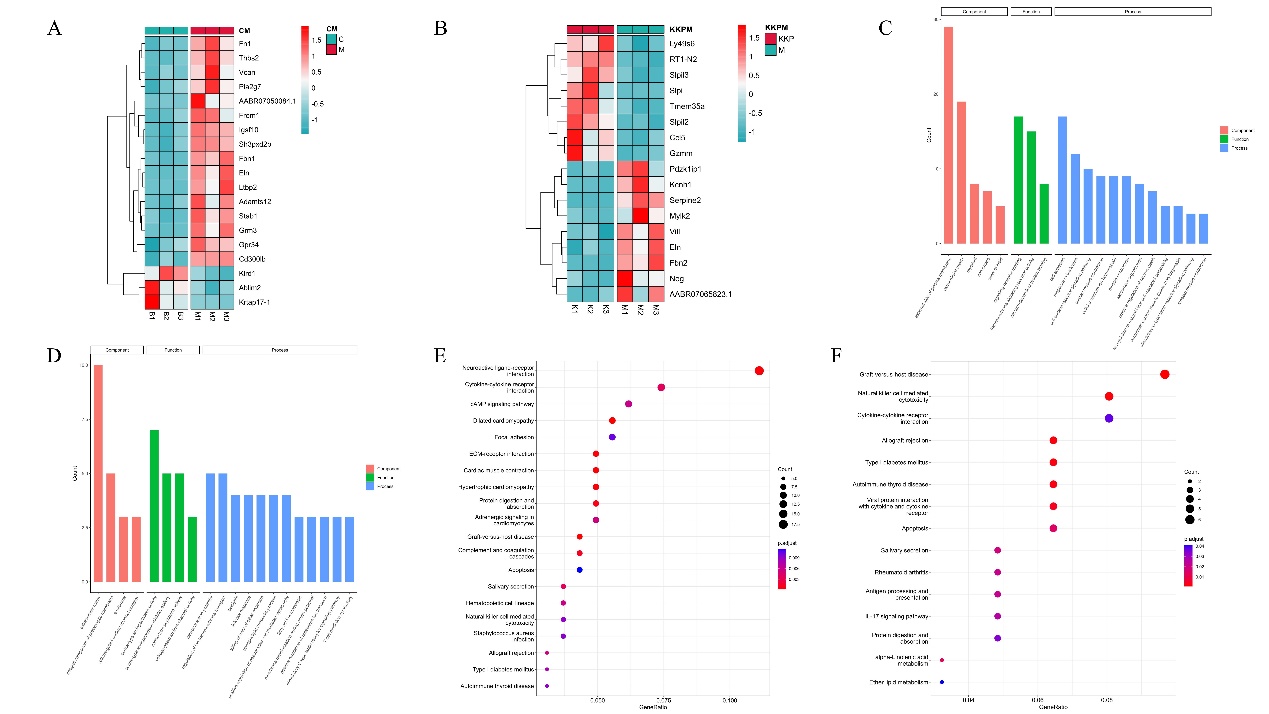


**Fig.S2.** (A) Statistical histogram of identification and quantitative results. (B) GO enrichment analysis for DEPs of Control vs. Model. (C) GO enrichment analysis for DEPs of KKP vs. Model. (D) KEGG enrichment analysis for DEPs of Control vs. Model. (E) KEGG enrichment analysis for DEPs of KKP vs. Model.


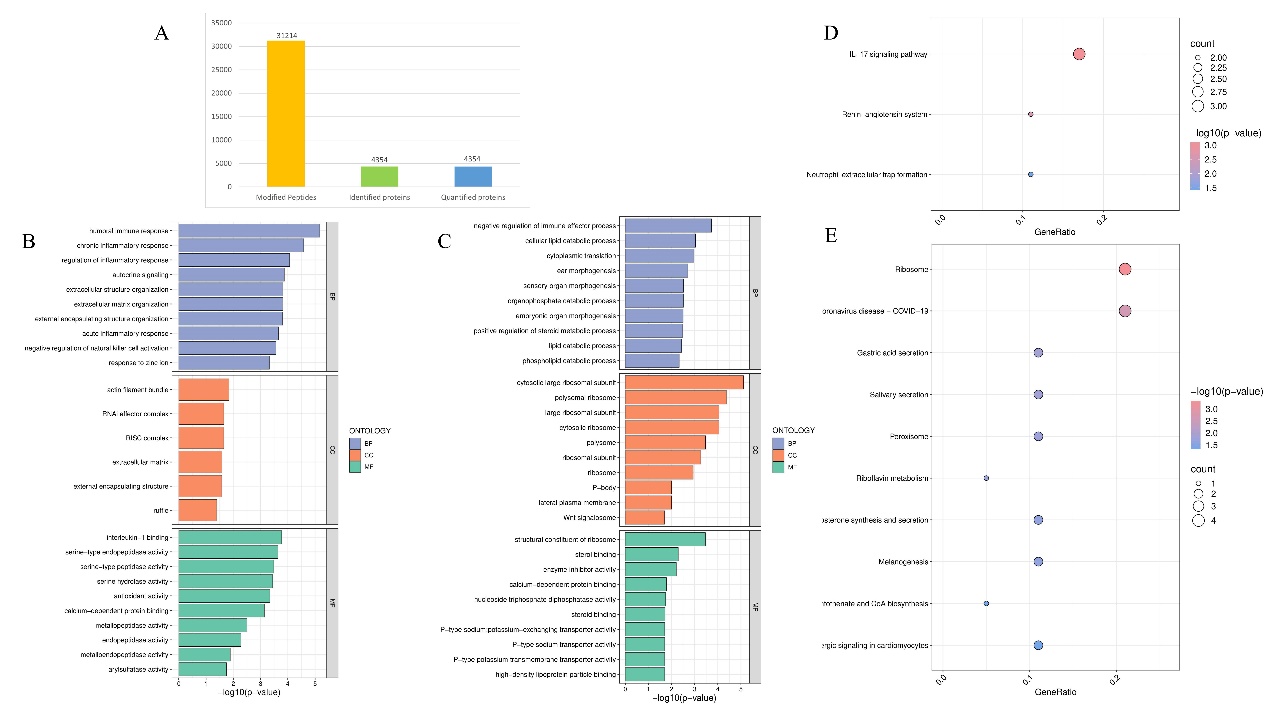


Table.S1. Mass spectrometric data and the identification results of constituents in KKP

| No. | t_R_ /min | Formula | [M-H]^+^/[M-H]^-^ | Error/ppm | Fragment irons(m/z) | Identification | Source |
| --- | --- | --- | --- | --- | --- | --- | --- |
| 1 | 1.052 | C_5_H_6_O_5_ | 175.1185/- | -2.86/- | （+）175，130，116，70 | Arginine | - |
| 2 | 1.132 | C_6_H_12_O_7_ | -/195.0511 | -/5.13 | （-）164，142，100，59，44 | Gluconicacid | JG，GC，LFZ，KXR |
| 3 | 1.135 | C_5_H_13_NO | 104.1061/- | -8.64/- | （+）104，60，45 | Choline | MH，LFZ，KXR，JG |
| 4 | 1.135 | C_5_H_9_NO_2_ | 118.0864/- | 0.85/- | （+）72，58，55，44 | Betaine* | - |
| 5 | 1.132 | C_5_H_7_NO_3_ | -/377.0859 | -/0.8 | （-）377，341，179，89 | Sucrose* | JG，GC |
| 6 | 1.135 | C_12_H_22_O_11_ | 116.0709/- | 2.58/- | （+）70 | Proline | GC，KXR，JG |
| 7 | 1.218 | C_5_H_11_NO_2_ | 130.0865/- | -1.54/- | （+）105 | Piperidinecarboxylicacid | LFZ |
| 8 | 1.466 | C_6_H_11_NO_2_ | -/191.0192 | -/-2.62 | （-）173，154，129，111 | Citricacid | GC，MH |
| 9 | 1.636 | C_7_H_13_NO_2_ | 144.1005/- | -9.7/- | （+）84，56 | Stachydrine* | GC |
| 10 | 1.719 | C_6_H_14_N_4_O_2_ | 130.0503/128.0353 | 3.08/117.16 | （+）109，84，56（-）125，82，74，55 | Pyroglutamicacid | JG，GC，LFZ，KXR，MH |
| 11 | 2.053 | C_13_H_16_O_9_ | 132.0987/- | -24.2/- | （+）131，90，86，57 | Leucine | GC，MH，KXR，JG |
| 12 | 2.303 | C_9_H_13_NO | 294.1555/- | 2.72/- | （+）276，258，230，123，86 | Fructosylisoleucineorisomer | LFZ，KXR，JG |
| 13 | 2.554 | C_9_H_13_NO | 268.1028/- | -4.48/- | （+）136，119 | Adenosine | MH，LFZ，KXR |
| 14 | 3.054 | C_6_H_13_NO_2_ | 166.123/- | 2.41/- | （+）121，103，93，91，77 | Hordenine* | MH |
| 15 | 3.972 | C_10_H_15_NO | 286.1433/- | -1.75/- | （+）286，268，181，165，153 | Morphine* | YSK |
| 16 | 4.974 | C_10_H_15_NO | 152.1064/- | -3.94/- | （+）134，115，91，56 | Norephedrine | MH |
| 17 | 5.558 | C_12_H_23_NO_7_ | 152.1061/- | -5.92/- | （+）134，117，115 | Norpseudoephedrine | MH |
| 18 | 6.726 | C_17_H_21_NO_3_ | 166.123/- | 2.41/- | （+）148，133，117，91 | Ephedrine* | MH |
| 19 | 7.474 | C_10_H_13_N_5_O_4_ | -/315.0719 | -/-0.95 | （-）246，201，153，109 | Protocatechuicacid-glucosideorisomer | MH |
| 20 | 7.727 | C_13_H_16_O_9_ | 166.1226/- | 0/- | （+）148，132，115，91，65 | Pseudoephedrine* | MH |
| 21 | 8.058 | C_10_H_15_NO | -/315.0726 | -/1.27 | （-）271，238，153，109，82 | Protocatechuicacid-glucosideorisomer | MH |
| 22 | 8.729 | C_11_H_17_NO | 180.1395/- | 6.66/- | （+）162，147，117，91，46 | Methylephedrine/Methylpseudoephedrine | MH |
| 23 | 9.98 | C_10_H_10_O_5_ | 328.1754/- | 0/- | （+）166，148，133，117 | Ephedrine-glucoside | MH |
| 24 | 9.644 | C_16_H_25_NO_6_ | -/209.0454 | -/-4.78 | （-）190，165，121，77，59 | 2-（4-Hydroxyphenyl）succinicacid | GC |
| 25 | 11.232 | C_8_H_12_N_2_ | 137.1058/- | -10.94/- | （+）110，80，55 | Tetramethylpyrazine | MH |
| 26 | 11.482 | C_20_H_30_N_2_O_8_ | 427.2066/- | -2.11/- | （+）265，177，145，117，89 | Feruloylputrescine-O-glucoside | MH |
| 27 | 11.566 | C_20_H_28_O_13_ | 494.186/475.1466 | -1.62/1.89 | （+）177，427（-）431，269，161，113 | Mandelicacid-β-gentiobioside | KXR |
| 28 | 12.066 | C_15_H_14_O_6_ | 300.1594/- | 0/- | （+）215，165，58 | Codeine* | YSK |
| 29 | 14.319 | C_18_H_21_NO_3_ | 493.2809/- | -0.81/- | （+）464，419，348，251 | EphedradineA | MH |
| 30 | 14.403 | C_28_H_36_N_4_O_4_ | 291.0863/289.0729 | -2.4/3.8 | （+）273，259，207，165，139，123（-）271，245，203，165，109 | Catechin* | MH |
| 31 | 15.821 | C_19_H_28_O_11_ | 222.1124/- | -0.45/- | （+）193，179，164，132，44 | Hydrocotarnine | YSK |
| 32 | 15.835 | C_12_H_15_NO_3_ | 450.1971/477.1617 | 0.22/0.63 | （+）325，253，217，163，145，91（-）431，269，161，159，101 | Benzyl-β-gentiobioside | KXR |
| 33 | 15.571 | C_22_H_32_O_13_ | 522.2183/549.1825 | 0.38/0 | （+）163，131，103（-）503，416，372，341，179，119 | Notoginsenicacid-β-sophoroside | MH |
| 34 | 16.489 | C_14_H_20_N_2_O_3_ | 265.1548/- | 0.38/- | （+）248，177，145，89 | Feruloylputrescine | MH |
| 35 | 16.737 | C_20_H_27_NO_11_ | 475.1925/456.1516 | 0.63/1.1 | （+）325，163，145，127，85（-）402，323，263，179 | L-Amygdalin* | KXR |
| 36 | 17.071 | C_20_H_27_NO_11_ | 475.1923/456.1516 | 0.21/1.1 | （+）325，163，145，127，85（-）376，323，294，221 | D-Amygdalin | KXR |
| 37 | 17.74 | C_14_H_17_NO_6_ | 318.0948/340.1045 | 0/2.06 | （+）290，260，233，217，122（-）317，234，181，161 | Prunasin | KXR |
| 38 | 17.836 | C_23_H_34_O_14_ | 552.2275/579.1946 | -2.17/2.59 | （+）193，161（-）533，341，179，143，89，44 | Siringinoside | MH |
| 39 | 18.74 | C_16_H_22_O_7_ | 344.17/371.1355 | -1.16/1.89 | （+）180，165，107，85（-）325，247，163，101，44 | Eugenylglucoside | MH |
| 40 | 18.832 | C_17_H_24_O_9_ | 390.1759/417.1402 | 0/0 | （+）211，193，180，161（-）257，207，176，98，44 | Syringin | MH |
| 41 | 19.66 | C_18_H_24_O_12_ | 316.1535/- | -2.53/- | （+）285，178，137，115 | Norreticuline | YSK |
| 42 | 21.32 | C_18_H_21_NO_4_ | 433.134/431.12 | -0.23/1.16 | （+）145，127（-）329，243，125，57 | LicoagrosideB | GC |
| 43 | 21.82 | C_13_H_25_O_8_ | -/547.1666 | -/-0.37 | （-）502，411，341，244，205，126 | SibiricoseA1 | LFZ |
| 44 | 22.747 | C_23_H_32_O_15_ | 310.1608/354.1565 | 4.51/9.6 | （+）251，207，175，147，119，91（-）333，294，279 | Sinapinethiocyanate* | LFZ |
| 45 | 23.24 | C_27_H_32_O_14_ | 598.2126/579.1724 | 0.67/0.86 | （+）419，257（-）417，342，255 | Liquiritigenin-7，4'-diglucoside | GC |
| 46 | 23.916 | C_19_H_21_NO_4_ | 328.1541/- | 0.61/- | （+）297，282，265，23 | Boldine | YSK |
| 47 | 25.167 | C_27_H_30_O_15_ | 342.1698/- | 0.58/- | （+）297，282，265，237，58 | Isocorypalmine | YSK |
| 48 | 26.085 | C_20_H_23_NO_4_ | 595.1655/593.1518 | -0.34/1.01 | （+）577，559，511，475，457，427，409（-）473，383，353 | Vicenin-2 | MH |
| 49 | 26.25 | C_23_H_32_O_15_ | -/547.169 | -/4.02 | （-）537，499，479，459，404，369 | Sinapicacid-gentiobioside | LFZ |
| 50 | 26.252 | C_26_H_28_O_14_ | 330.1694/- | -1.82/- | （+）299，192，175，137 | Reticuline | YSK |
| 51 | 26.336 | C_26_H_28_O_14_ | 400.1385/- | -1.5/- | （+）351，339，206 | Narcotoline | YSK |
| 52 | 27.42 | C_19_H_23_NO_4_ | 328.1541/- | -0.61/- | （+）178，163，151 | Scoulerine | YSK |
| 53 | 27.671 | C_21_H_21_NO_7_ | 565.1545/563.1409 | -1.24/0.53 | （+）547，529，475 | Schaftoside | GC |
| 54 | 28.255 | C_19_H_21_NO_4_ | 565.1545/563.1409 | -1.24/0.53 | （+）547，529，511，481，427（-）503，473，443，383，353 | Isoschaftoside | GC |
| 55 | 28.586 | C_21_H_22_O_9_ | 344.1856/- | 0/- | （+）313，192，151，115 | Laudanine | YSK |
| 56 | 25.589 | C_20_H_25_NO_4_ | 419.1337/417.1191 | 0/- | （+）257，211，137（-）255，135，119 | Neoliquiritin |  |
| 57 | 28.672 | C_21_H_22_O_9_ | 312.1597/- | 0.96/- | （+）251，58 | Thebaine | YSK |
| 58 | 29.087 | C_21_H_20_O_10_ | -/417.1191 | -/0 | （-）255，135，119，91 | Liquiritin | GC |
| 59 | 29.506 | C_26_H_30_O_13_ | 344.1856/- | 0/- | （+）313，206，189，137 | Codamine | YSK |
| 60 | 29.673 | C_21_H_20_O_10_ | 433.1122/- | 1.62/- | （+）415，397，337，313，283 | Vitexin | MH |
| 61 | 29.757 | C_27_H_30_O_14_ | 326.1395/- | 2.45/- | （+）310，188，156，128 | Palaudine | YSK |
| 62 | 29.838 | C_19_H_21_NO_3_ | -/549.1614 | -/1.82 | （-）386，297，255，135 | Liquiritinapioside | GC |
| 63 | 30.423 | C_20_H_25_NO_4_ | 431.0988/- | 1.62/- | （-）413，341，311 | Isovitexin | MH |
| 64 | 30.591 | C_19_H_19_NO_4_ | 579.1718/577.1563 | 1.73/0 | （+）433，415，397，379，367（-）413，341，293 | Isoviolanthin | MH |
| 65 | 31.257 | C_9_H_16_O_4_ | -/187.0976 | －/0 | （-）169，125，97，71 | Azelaicacid* | - |
| 66 | 31.259 | C_16_H_12_O_6_ | 354.1333/- | -0.85/- | （+）334，312，306 | Protopine | YSK |
| 67 | 31.426 | C_34_H_42_O_19_ | 370.1643/- | -1.62/- | （+）352，339，336，320 | Cryptopine | YSK |
| 68 | 31.759 | C_20_H_19_NO_5_ | 358.2009/- | -1.12/- | （+）327，312，296 | Laudanosine | YSK |
| 69 | 32.51 | C_21_H_23_NO_5_ | 301.0701/- | -2/- | （+）301 | 3-Methylkaempferol | GC |
| 70 | 33.762 | C_21_H_27_NO_4_ | 414.1552/- | 1.21/- | （+）396，365，353，323 | Narcotine | YSK |
| 71 | 33.846 | C_22_H_23_NO_7_ | 340.155/- | -2.06/- | （+）324，308，296 | Papaverine* | YSK |
| 72 | 34.595 | C_20_H_21_NO_4_ | -/753.2252 | -/0.53 | （+）207，175，147，119，91，65（-）463，753，816 | 3'，6-Disinapoylsucrose | LFZ |
| 73 | 34.93 | C_22_H_22_O_9_ | 446.1813/- | 0.9/- | （+）428，383，365，350 | Narcein | YSK |
| 74 | 35.514 | C_23_H_27_NO_8_ | 431.1333/475.125 | -0.93/0.84 | （+）269，237（-）355，267，207，175，106 | Formononetin-7-O-β-D-glucoside | GC |
| 75 | 35.598 | C_26_H_30_O_13_ | 551.1763/549.1624 | 0.73/1.82 | （+）419，257，211，147，137（-）417，255，135 | Isoliquiritinapioside* | GC |
| 76 | 35.765 | C_15_H_12_O_4_ | 257.0814/255.0669 | 2.33/2.35 | （+）239，147，137（-）202，135，119 | Liquiritigenin* | GC |
| 77 | 35.846 | C_21_H_22_O_9_ | 419.1337/417.1191 | 0/0 | （+）257，137，81（-）255，148，135，92 | Isoliquiritin* | GC |
| 78 | 36.432 | C_16_H_14_O_5_ | 287.0926/285.0776 | 4.18/2.81 | （+）245，193，150，121（-）270，247，150 | LicochalconeB* | GC |
| 79 | 38.076 | C_16_H_12_O_5_ | 285.0758/- | 0.35/- | （+）270，253，225，213，137 | Calycosin* | GC |
| 80 | 42.272 | C_18_H_32_O_5_ | -/895.3936 | -/-3.69 | （-）729，641，519，411，351，193 | 22β-AcetoxylicoricesaponinG2orisomer | GC |
| 81 | 42.438 | C_44_H_64_O_19_ | 346.2589/327.2179 | 0.29/0.61 | （-）291，229，221，211，171 | 9，12，13-Trihydroxy-10，15-octadecadienoicacidorisomer | - |
| 82 | 42.524 | C_48_H_72_O_21_ | 354.1332/- | -1.13/- | （+）265，188，157，128 | Papaveraldine | YSK |
| 83 | 43.192 | C_20_H_19_NO_5_ | 985.4641/983.4503 | 0.2/1.02 | （+）809，647，615，453，407（-）821，351，193，61 | Licorice-saponinA3orisomer | GC |
| 84 | 43.523 | C_15_H_12_O_4_ | 257.0814/255.0669 | 2.33/2.35 | （+）242，211，147，137，81（-）135，119，93 | Isoliquiritigenin | GC |
| 85 | 43.859 | C_16_H_12_O_4_ | 269.0801/267.0672 | -2.6/3.37 | （+）253，226，213，197，181，175（-）252，224，201，136，100 | Formononetin | GC |
| 86 | 45.445 | C_42_H_62_O_17_ | 839.4075/837.3927 | 1.79/1.56 | （+）663，645，487，469，451（-）775，661，351，193 | 22-Hydroxyl-glycyrrhizinorisomer | GC |
| 87 | 46.029 | C_42_H_62_O_17_ | 839.4067/837.3916 | 0.83/0.24 | （+）645，487，469，451（-）775，619，469，351，286 | 22-Hydroxyl-glycyrrhizinorisomer | GC |
| 88 | 46.363 | C_42_H_62_O_17_ | 839.4071/837.3934 | 1.31/2.39 | （+）663，645，487，469，451（-）661，351，193 | 22-Hydroxyl-glycyrrhizinorisomer | GC |
| 89 | 46.696 | C_42_H_62_O_16_ | 823.4111/821.3965 | 0/0 | （+）647，453，357（-）759，645，351，193 | Glycyrrhizicacid | GC |
| 90 | 47.614 | C_21_H_22_O_8_ | 403.1386/- | -0.25/- | （+）388，373，358，345 | Nobiletin* | JG |
| 91 | 47.698 | C_42_H_62_O_16_ | 823.4111/821.3965 | 0/0 | （+）757，647，453（-）759，351，193 | Glycyrrhizicacidisomer | GC |
| 92 | 48.783 | C_42_H_64_O_16_ | 825.4287/- | 2.42/- | （+）749，564，455，437，317，141 | LicoricesaponinJ2orisomer | GC |
| 93 | 48.699 | C_21_H_20_O_6_ | 369.1335/- | 0.54/- | （+）313，285，270 | Glycycoumarin | GC |
| 94 | 49.367 | C_20_H_20_O_7_ | 373.1277/- | -1.34/- | （+）358，343，271，179 | Tangeretin | JG |
